# Supplementary figures and images for: Dynamics of the cell fate specifications during female gametophyte development in Arabidopsis
Source: PLoS Biol. 2021 Mar 26;19(3):e3001123. doi: 10.1371/journal.pbio.3001123 (PMC7997040; doi:10.1371/journal.pbio.3001123)

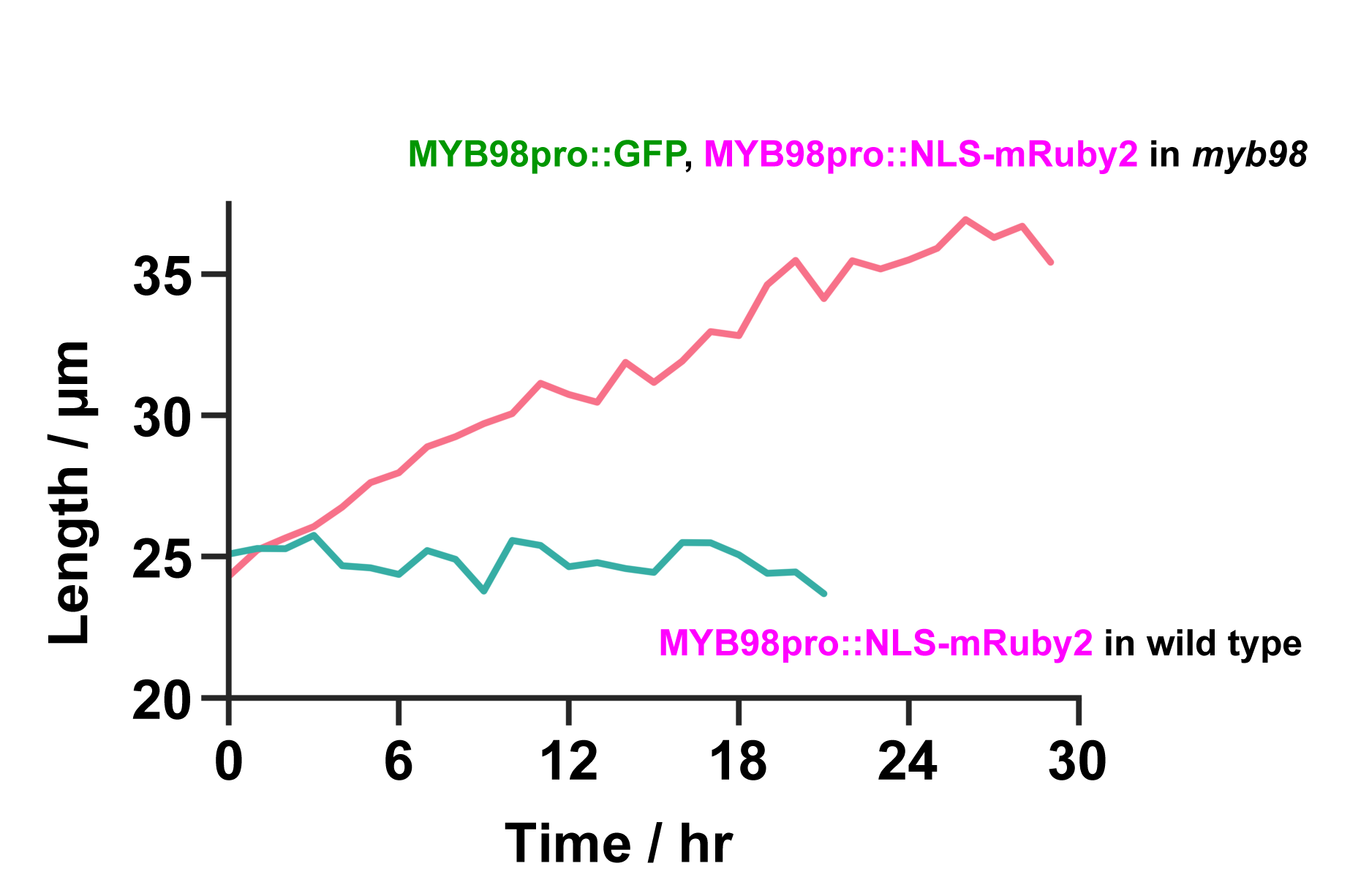

Supplement: S1 Fig — We measured the longitudinal length of the synergid cells every 1 hour for MYB98pro::NLS–mRuby2 in a wild-type ovule and MYB98pro::NLS–mRuby2 and MYB98pro::GFP in a myb98 ovule from S8 and S9 Movies. The underlying numerical data for this figure can be found in S1 Data. (TIF) [file pbio.3001123.s001.tif]

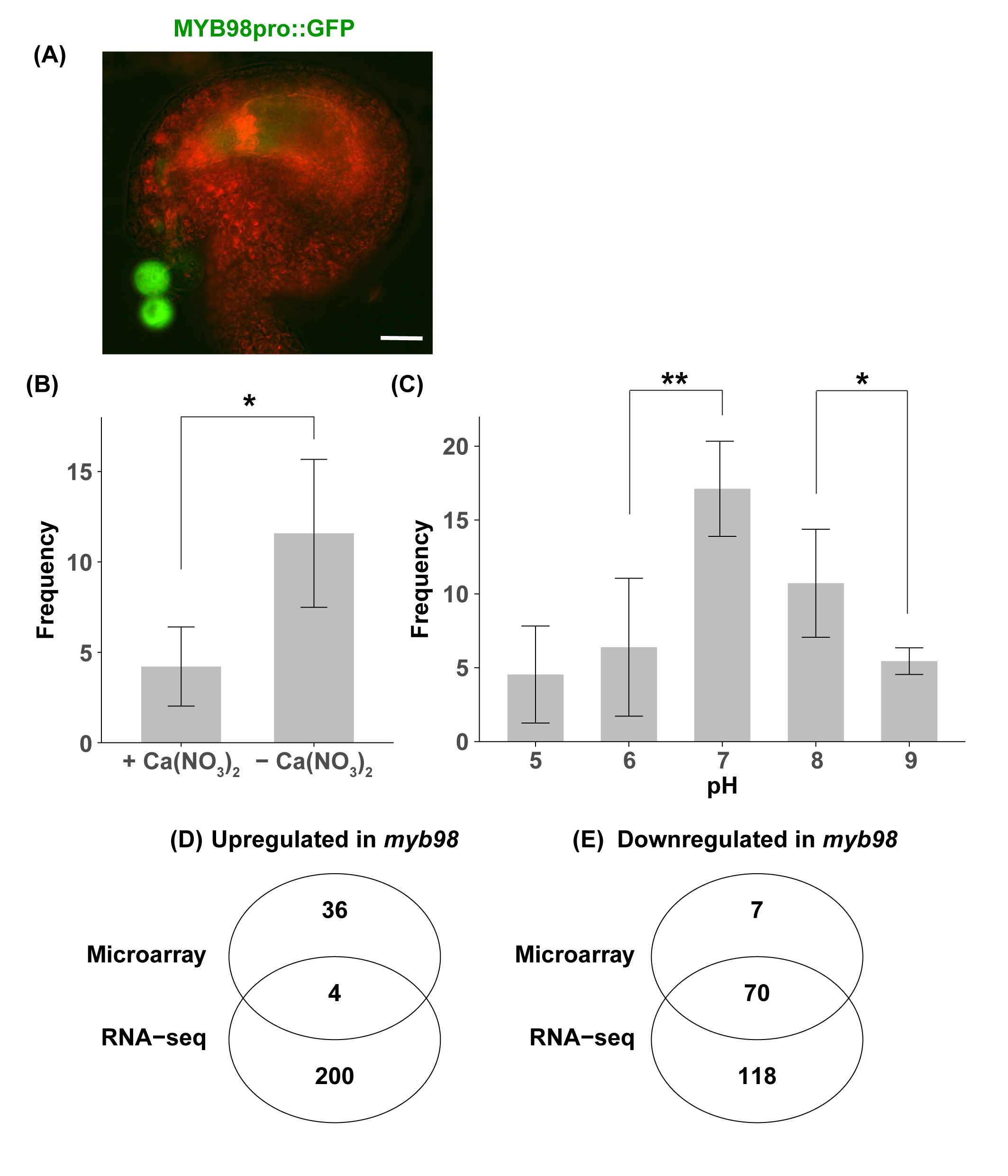

Supplement: S2 Fig — (A) Two synergid cells were released from the ovules of MYB98pro::GFP. (B) Frequency of the collectable synergid cells with or without calcium nitrate in the enzyme solution. An F-test of the frequency showed that the variances of the groups with and without calcium nitrate were equal (p > 0.05). The results of Student t test showed that the absence of calcium nitrate was more effective for synergid cell isolation (*p < 0.05). (C) Frequency of the collectable synergid cells depending on the pH of the enzyme solution. An F-test of the frequency showed that the variances between pH 6 and pH 7 and pH 7 and pH 8 were equal (p > 0.05). A result of Student t test showed that the frequency of collectable synergid cells was different significantly between pH 6 and pH 7 and pH 7 and pH 8(*p < 0.05; **p < 0.01). (C, D) Venn diagram of DEGs that were up-regulated (D) or down-regulated (E) in the myb98 mutant synergids between the RNA-seq and microarray. The underlying numerical data for (B, C) can be found in S1 Data. DEG, differentially expressed gene; RNA-seq, RNA sequencing. (TIF) [file pbio.3001123.s002.tif]

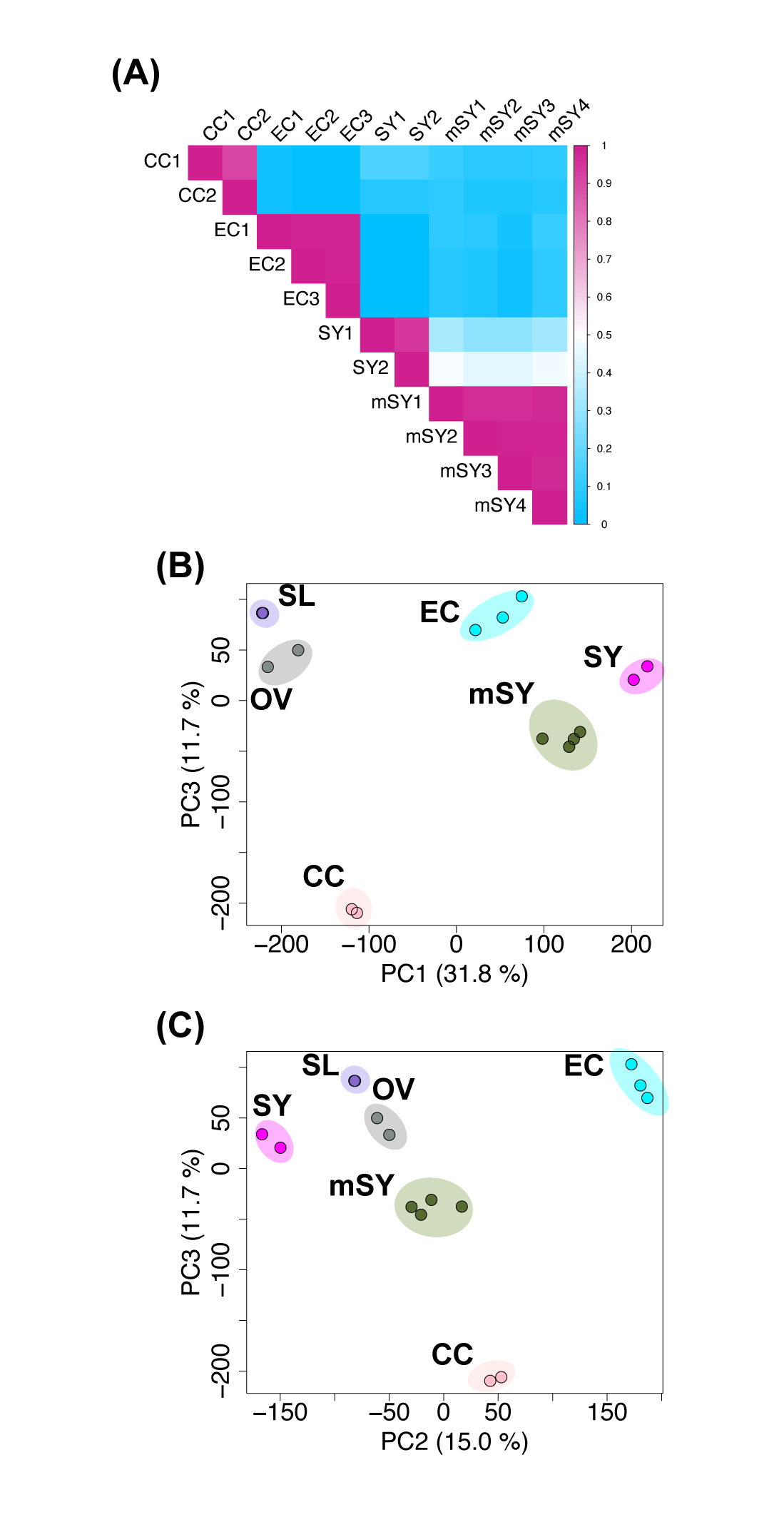

Supplement: S3 Fig — (A) The Pearson correlation of RNA-seq libraries. (B, C) The PCA analysis (PC1 vs. PC3, PC2 vs. PC3) of all transcriptome data. The underlying numerical data for this figure can be found in S1 Data. PCA, principal component analysis; RNA-seq, RNA sequencing. (TIF) [file pbio.3001123.s003.tif]

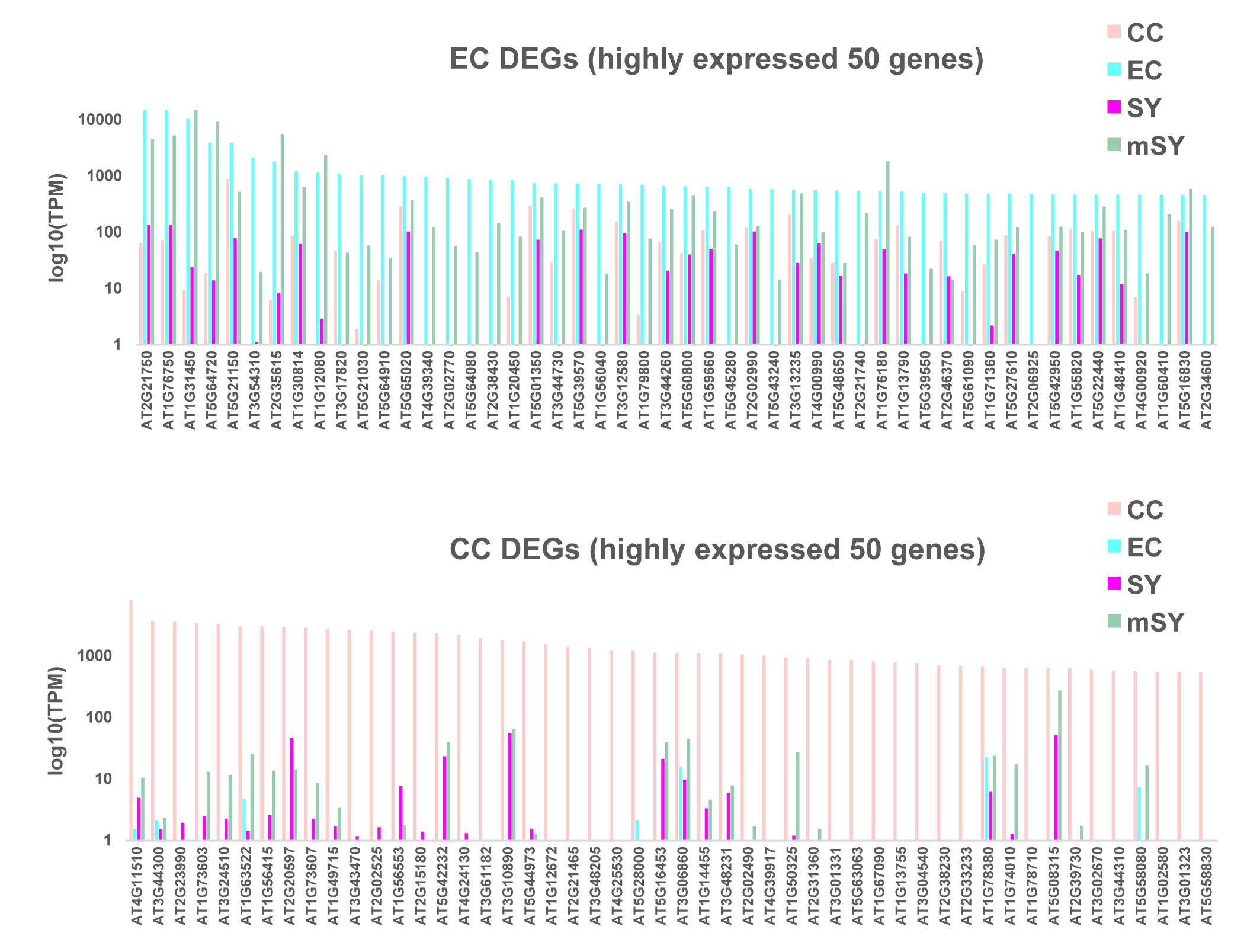

Supplement: S4 Fig — Highly expressed genes of myb98 synergid cells were more abundant in DEGs of egg cells than those of central cells. The underlying numerical data for this figure can be found in S1 Data. DEG, differentially expressed gene. (TIF) [file pbio.3001123.s004.tif]

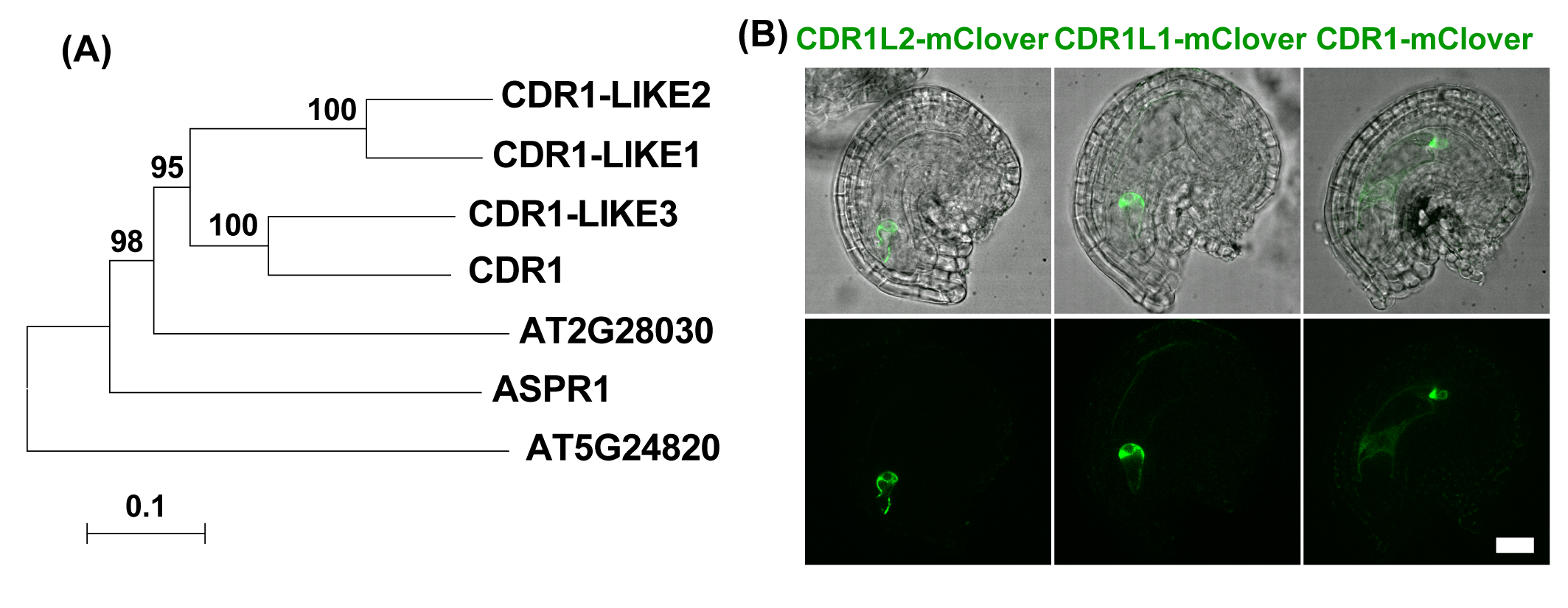

Supplement: S5 Fig — (A) Phylogenetic tree of the aspartyl proteases in the Arabidopsis thaliana. (B) The expression patterns of the CDR1L2–mClover and CDR1L1–mClover were detected in the egg cell. The fluorescent signal of the CDR1–mClover was detected in the central cell and the antipodal cells. Scale bar: 20 μm. (TIF) [file pbio.3001123.s005.tif]

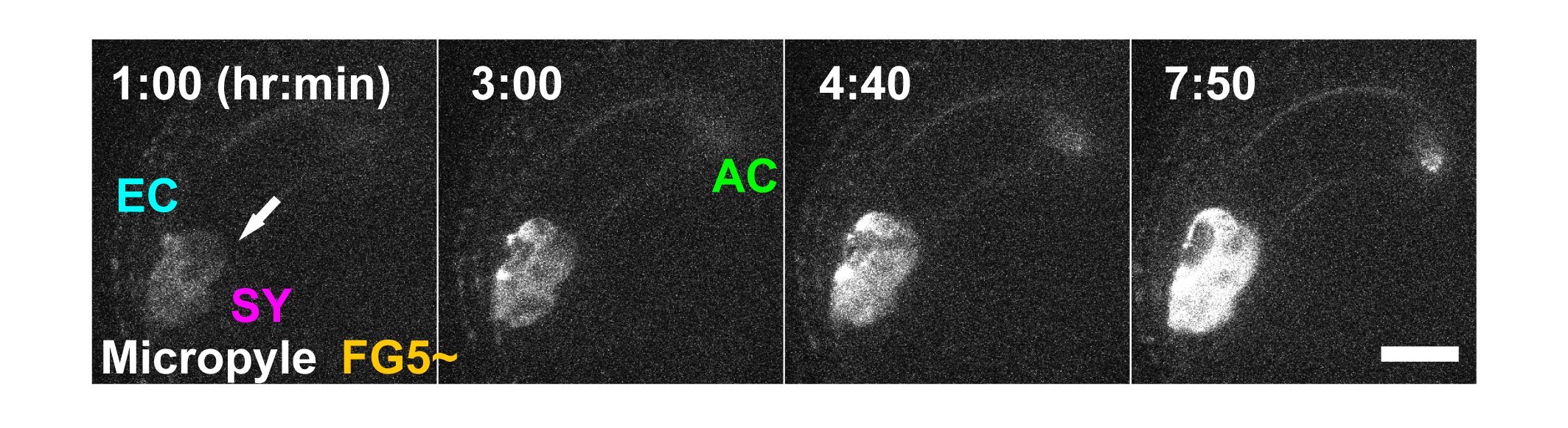

Supplement: S6 Fig — The numbers indicate the time (hr:min) from the first detection of the SBT4.13–mClover. The fluorescent signals of the SBT4.13–mClover were also detected in the synergid cells and the antipodal cells. Scale bar: 20 μm. (TIF) [file pbio.3001123.s006.tif]

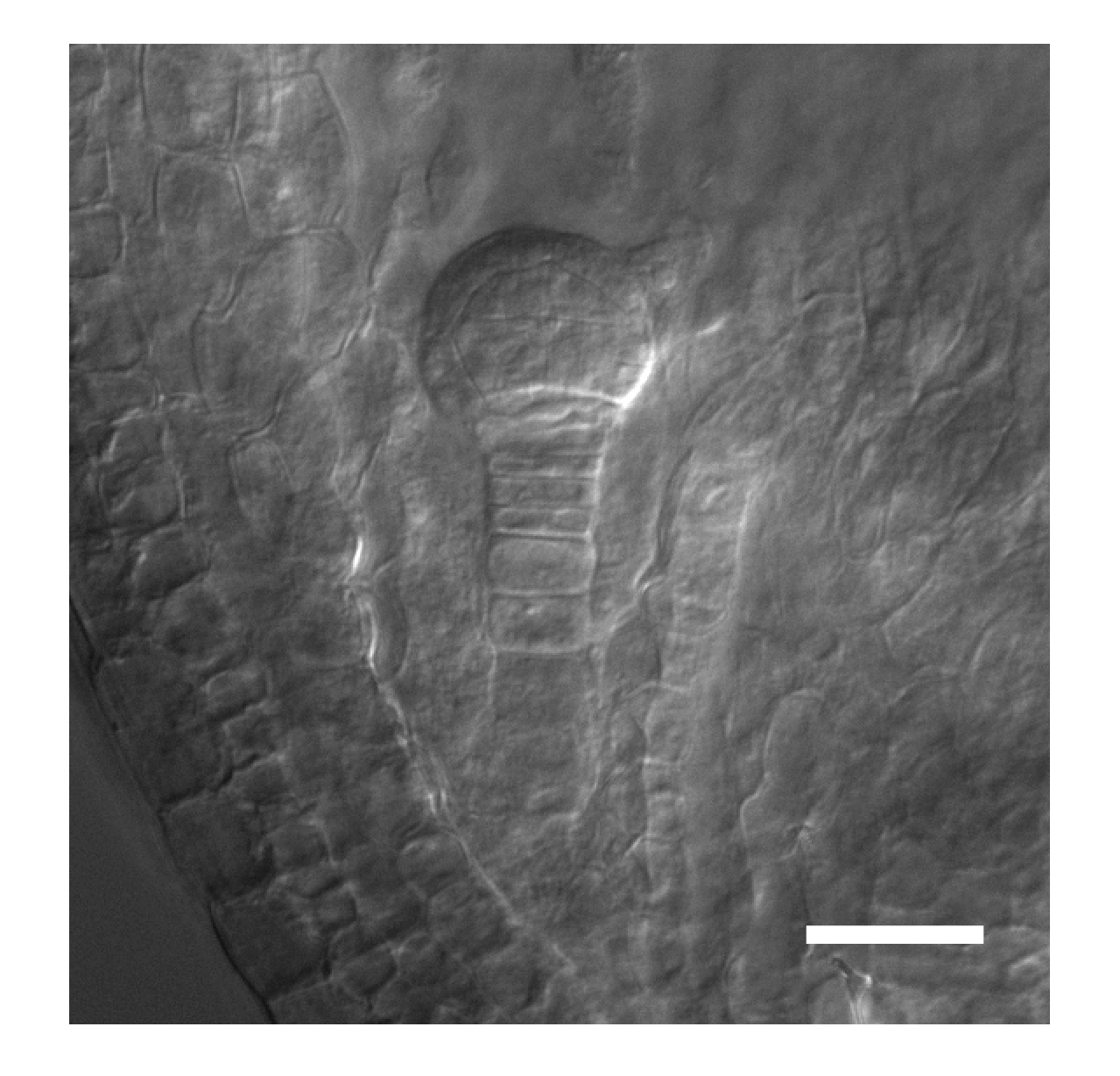

Supplement: S7 Fig — DIC images showed the cleared globular embryos of the myb98 mutant. Scale bar: 20 μm. DIC, disseminated intravascular coagulation. (TIF) [file pbio.3001123.s007.tif]
