# Supplementary material for: Dynamics of the cell fate specifications during female gametophyte development in Arabidopsis
Source: PLoS Biol. 2021 Mar 26;19(3):e3001123. doi: 10.1371/journal.pbio.3001123 (PMC7997040; doi:10.1371/journal.pbio.3001123)
Supplement: Attachment — Submitted filename: 105727_1_rebuttal_1801451_qd3wzt.pdf [file pbio.3001123.s037.pdf]

Point-by-Point Response to Reviewer #1

Major comments

We thank you for your kind reviewing and helpful comments. As with your advice, we will improve our manuscript with the following points.

Point 1

1) In wild type they note early detection of MYB98pro::GFP throughout the gametophyte prior to cellularization and rapid activation of the EC genes in the newly formed egg cell. However, an important caveat is in the pre-cellularized state (the coenocytic gametophyte), the movement of free GFP, and that of NLS-mRuby2 prior to nuclear import, are both likely and possible respectively. Thus, marker expression will not necessarily mirror localization of MYB98 protein prior to cellularization. Moreover, the longevity of the relatively stable fluorescent proteins could lead to the appearance of expression in newly formed egg and central cells.

Response: As Reviewer#1 comment, that's certainly a possibility. As mentioned in another comments, I think it would be useful to observe the MYB98–fusion fluorescent protein. We are growing the seeds of MYB98pro::MYB98–GFP now and will need two more months to observe and analyze them.

Point 2

2) They find that the position of nuclei in the synergids after cellularization is not maintained in myb98 mutants and nuclear positioning becomes more like that in the egg cell. This leads to a muddled message about the role of nuclear position leading to cell fate (and gene expression) vs. gene expression leading to nuclear position: Nuclear position regulates MYB98 initiation but, in myb98, nuclei move around; later MYB98 is still expressed despite this nuclear movement. This apparent contradiction should be more clearly discussed and possible explanations provided.

Response: As shown in Figure 4B, the nucleus moves around the center of the synergid cells in myb98 mutant. Therefore, we believe that myb98 synergids can express the specific genes of both egg cell and synergid cell. mSY forms a separate cluster from EC and SY in the PCA analysis (Fig. 6C), suggesting that this possibility. We will discuss this possibility in the revised manuscript.

Point 3

3) The role of MYB98 in cell fate is investigated by comparing wild type and mutant synergid transcriptomes and cell type marker expression. The authors show that some egg-specific genes are expressed in synergids using transgenes. They conclude from their transcriptomic analysis that mutant synergids are more similar to egg cells.

a. Their RNAseq analyses are not sufficiently well described for full understanding of the results, as detailed in minor comments below. Importantly for low-input RNA libraries, the authors should describe the correlation between replicates statistically, and strategies for overcoming amplification error.

Response: We will add the detail in RNA-seq libraries and describe the correlation between the replicates.

b. It appears that their experiments showed both egg and central cells genes are expressed in mutant synergids. From Figure 6A: 225 egg specific genes are found in the myb98 synergid (22% of egg specific genes). However, there are an even greater number of central cell genes mis-expressed in mutant synergids: 838 central cell genes that are turned on (15% of central cell specific genes). Even when only DEGs (SY vs mSY) are used (Figure 6D) and from looking at Table S3 (21 egg genes vs 17 CC genes), there are clearly a comparable number of CC genes expressed in mutant synergids. These central cell expressed genes are not sufficiently addressed in the text. The authors choose to test only egg cell genes by promoter-reporter constructs. The somewhat closer clustering of mutant synergids with egg cells is an indication that overall the cells are more egg cell-like but if that is the most important piece of evidence there should be more details about how the analysis was done. Given the other points above, other interpretations should be considered.

Response: As Reviewer#1 suggests, we will improve the method in the manuscript for the hierarchical clustering analysis as follows: ”According to the value of the Spearman correlation between different samples, hierarchical clustering analysis was carried out using a phylogram package (`https://github.com/rambaut/figtree/`). We investigated the egg cell-specific genes because the clade of *myb98* synergid cell was combined into the same clade as the egg cell, not the synergid cell clade in Hierarchical clustering (Fig. 6B).”

We show the gene expression of highly expressed 50 DEGs in egg cells and central cell in the following figure. Highly expressed genes of *myb98* synergid cells were more abundant in DEGs of egg cells than those of central cells. Therefore we focused on the egg cell-specific genes.

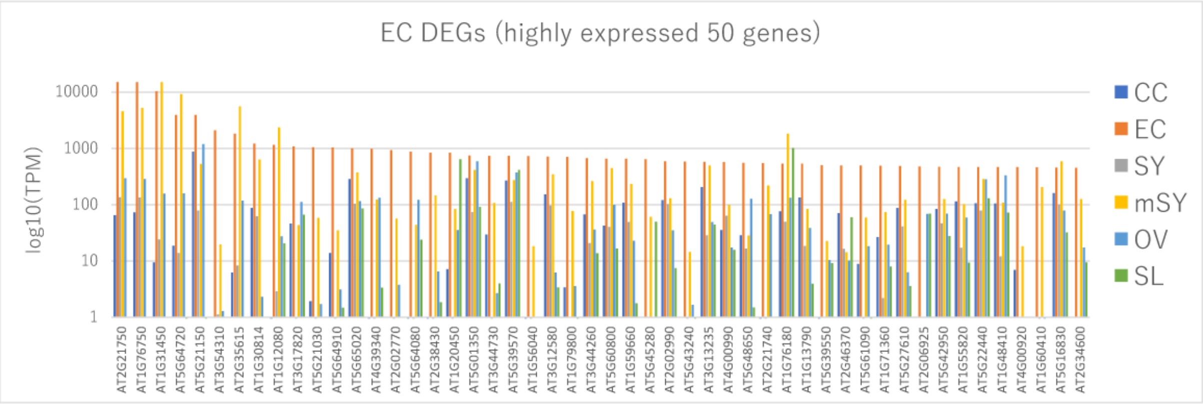

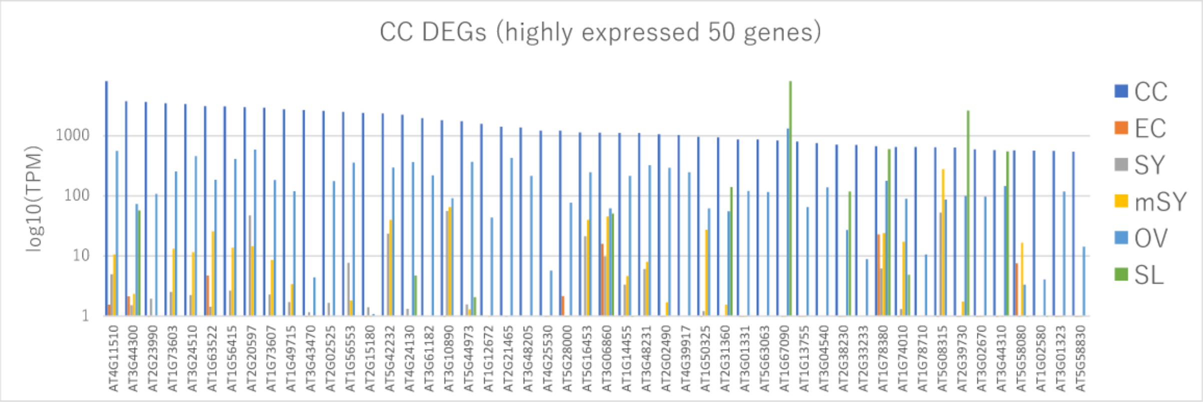

c. There is little discussion of the actual genes that are changed in expression in the myb98 mutant. Do these provide any evidence of acquisition of cell fate, such as increased expression of egg-cell transcription factors (RKD family) or other regulation?

Response: In our study, RKD1 and RKD2 and RKD2–induced genes were identified as egg cell DEGs, *myb98* DEGs (Köszegi et al., 2011). We confirmed the expression levels of them in textitmyb98 synergid cells. RKD1 and RKD2 were upregulated in a replicate of *myb98* synergid cells. RKD2–induced genes tended to be up–regulated in *myb98* synergid cells. Moreover, EC1.5 was detected as a DEG in *myb98* synergid cell. Other EC1s also tended to be up–regulated in *myb98* synergid cells. We will improved the manuscript including above information.

Point 4

4) The authors state that the maintenance, not initiation, of synergid genes was defective in myb98. However, they only give early expression information for MYB98, and note that the synergids were phenotypically abnormal prior to maturity. No other synergid genes were examined to observe their expression dynamics. No discussion is made of synergid specific genes that were unaffected in the RNAseq of mutants. Some egg genes were observed just after cellularization, indicating an early change from synergid to egg-like. The conclusion that synergids are initiated correctly do not appear to be supported by the data presented.

Response: As Reviewer#1 comment, we only observed the MYB98 expression for the synergid specific genes. We intended that the initiation of synergid cell fate was unaffected, not that the initiation of expression of each synergid gene was unaffected. The subheading "The maintenance, not initiation, of synergid specific genes were defective in myb98" is misleading. So we will change to "The maintenance, not initiation, of synergid cell fate were defective in myb98".

Point 5

5) MYB98pro::reporter signals were observed throughout the gametophyte prior to and just after cellularization. The expression of one of three examined egg-specific genes (SBT4.13) was observed in antipodals 30% of the time. In contrast, two other genes were observed only in egg and synergid cells. From this, the authors suggest MYB98 negatively regulates egg cell fate in both synergids and antipodals.

a. As mentioned in the first comment, the authors do not account for the movement and persistence of fluorescent reporter proteins which means the true presence of MYB98 protein in antipodals is uncertain. A translational MYB98-GFP fusion should be used if the authors wish to conclude function from reporter expression.

Response: As a response to Point 1, we plant to observe MYB98pro::MYB98–GFP.

b. The number of examined ovules in Tables 1 and 2 (from 6 to 23 ovules) is much lower than typically used to determine trends in gene expression. If these are live imaging experiments then from each experiment, there might be multiple observations of the same ovule, which provides more confidence than a single observation, and might be the reason for the lower number observed. However, to be convincing on the specific points regarding gene expression in the mature FG6 gametophyte in the WT and mutant ovules, a more conventional analysis with greater numbers of observed ovules (n>50) is recommended.

Response: 6 to 23 ovules in Tables 1 and 2 mean different ovules, not the same ovules. But as Reviewer#1 comment, the sample size for the live-imaging experiment is small. We are also growing the seeds of CDR1–LIKE2 and SBT4.13 in WT and *myb98* to add the data of conventional analysis (need two more months).

Point 6

6) The authors state that there is usually conversion of only one of the synergids to an egg-like state and that this is similar to the known synergid-to-egg conversion when the egg is defective. The authors conclude that this indicates synergid-to-synergid communication. The evidence shown does not make clear that this is the case.

a. For CDR1-L2 and SBT4.13 markers, figures 7C and 8B do not show single synergids, and the information in Table 2 is insufficiently explained to be understood. If these are the same observations as described in Table 1 - what makes the two Tables different is quite unclear (aside from the obviously different numbers in each sub-category). If single synergids are being counted, then shouldn't the total should be double?

Response: We apologize that the label of Table 2 is a mistake. The correct label is shown in the table below.

Construct	MYB98 genotype	0 SY	1 SY	2 SY
CDR1–LIKE2pro::	+/+	6/6 (100 %)	0	0
CDR1–LIKE2–mClover	-/-	0	8/9 (89%)	1/9 (1%)
SBT4.13pro::	+/+	10/10 (100%)	0	0
SBT4.13–mClover	-/-	1/23 (4%)	17/23 (74%)	5/23 (22%)

Table 2 : Number of mutated synergid cells with EC-specific gene expressions

b. It is not stated how many times the EC1.1pro expression (Fig8C) was observed.

Response: We observed ten ovules of this line. Five ovules (50 %, n = 10) showed EC1.1pro expression in the synergid cell and remained five ovules showed WT-like.

c. Finally, even if this were clear, a simpler explanation would be that once the synergid acquires some egg-like characteristics, this may include the ability to signal to neighboring synergids to prevent another egg from developing as has been described for egg cells. In other words, what is being observed is another manifestation of the previously described egg cell to synergid communication, rather than synergid to synergid communication.

Response: There is certainly the possibility of communication from the newly egg cell to the synergid cell. In this case, it is necessary to acquire the egg cell characteristics, and there is a time lag before communication occurs. Because some egg-cell specific genes were detected just after cellularization, there should be more cases in which the fluorescent signals of EC-specific genes were detected in the two synergid cells simultaneously. So we think there is also communication between the synergid to synergid cell. We will also discuss the possibility of communication from newly egg cell to synergid cell in the revised manuscript.

Minor comments

Point 1

1) All figures and Supplemental movies It is important for each time-lapse experiment to clearly state in the legend or text the number of cells observed for each construct.

Response: We will provide the sample size in the legend and/or supplemental table.

Point 2

2) Results 3.1 Figure 1A - the model of nuclear movement does not match that described in the text, which is shown in Figure 9. This is confusing to the reader. Figure 1A could be replaced with a simpler version that only shows the stages and not nuclear movements or planes of division. The legend has C mislabeled as B.

Response: Considering the comments of all Reviewers, we will remove Fig. 1A.

Point 3

3) It would useful for the authors to compare the findings from Arabidopsis with their prior results on nuclear movement from Torenia (Susaki et al., 2015).

Response: As Reviewer #1 advice, we will improve the discussion about the nuclear movement to compare with the previous study in *Torenia*.

Point 4

4) Results 3.3 and beyond There is a bewildering array of promoters and reporters which make the manuscript very difficult to follow, especially as some of the promoters are from genes that are somewhat obscure. It is highly recommended that the authors provide a table to show different promoters, markers, cellular localization and their previously known stages of detection.

Response: We will make the marker lines list as a Supplemental Table.

Point 5

5) Line 15/16 ”in the ovules of the egg cells and the MYB98pro::GFP ovules of the synergid cells” This sentence needs editing, as the meaning is not clear.

Response: We will modified the sentence as follows: ”We observed the egg cell marker, EC1.2pro::mtKaede (Hamamura et al., 2011), and the synergid cell marker, MYB98pro::GFP (Kasahara et al., 2005) (Figure 3A,B, Supplementary Movie 5, 6)”.

Point 6

6) Line 24 ABI4 needs reference

Response: We will describe the reference for ABI4 (Wang et al., 2010) in the table of marker list.

Point 7

7) Figure 3B The black/white pictures of Myb98::GFP are somewhat fuzzy, and definitely not as clear as the colored images with the other marker.

Response: We will change Fig. 3B as the colored images.

Point 8

8) Figure 3E and Movie S8 There is a missing marker not noted on the figure and movie (purple sporophytic, not AKV), and AKV not labeled in Figure. The use of the non-contrasting sporophytic background marker makes this experiment hard to see clearly.

Response: To make a highly expressed nuclear marker in female gametophyte, 5' UTR of AtADH (Alcohol dehydrogenase) was inserted between AKV promoter and H2B coding sequence. Unfortunately, this AKVpro::H2B-mScarlet-I was expressed not only in female gametophyte but also in sporophytic cells. We will describe the construct in the material and methods.

Point 9

9) Results 3.4 Please indicate the number of observations made. Figure 4 should be all in color. If equivalent pictures of MYB98pro::GFP/NLS-Ruby in WT are available these should be used, as it is difficult to see the extent of the synergid in A.

Response: Unfortunately, we have not MYB98pro::GFP, MYB98pro::NLS–mRuby2 in Col. To make it easier to see, we will changed Fig. 4 as the colored images. Now, we try to transform the MYB98pro::GFP, MYB98pro::NLS–mRuby2 in Col., but three months are needed for analysis of T1 plants.

Point 10

10) Line 50 states that the myb98 synergids were more elongated but it is not possible to see this from the figure, nor are any other measurements given.

Response: We showed the measurement of longitudinal length in the *myb98* synergid cell of Supplemental Movie 10 as follows.

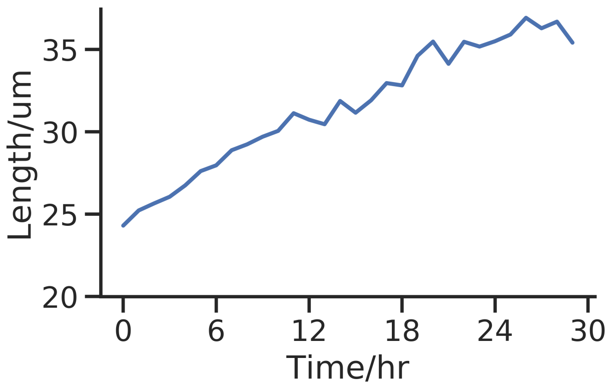

We will add the measurements like this graph as a Supplemental Figure.

Point 11

11) Results 3.5 Figure S1A. A bright field image is needed to show that there are no attached cell debris or other cells.

Response: We don't have the bright field image for Fig S1A. Instead, we will add the bright field images to Fig 5.

Point 12

12) Figure S1 B legend reads: "F test of frequency showed that there is no significant difference between the two variances ($p > 0.05$)." However, the figure has a single asterisk between the two columns which should indicate that there was a significant difference. RNAseq: With such few cells (12–18) it is important to show that reps are consistent, which was partially done using the PCA and clustering. However, more details are needed, including a correlation analysis. Did the authors use methods to reduce PCR duplicates which would be common from low-input libraries? How did the datasets compare with other cell-specific datasets (Schmid, 2012 and Zhou, 2019). Was there evidence of contamination between cell-types by looking at TPM of genes with known expression patterns?

Response: F-test is the process of checking if the variances of the two groups are equal. First, we confirmed by F-test that the variances of the groups with and without calcium nitrate were equal ($p > 0.05$). Next, Student's t-test showed the absence of calcium nitrate was more effective for synergid cell isolation (*$p < 0.05$). As a response to Point 3 in major comments, we will add the detail in RNA-seq libraries and describe the correlation between the replicates.

Point 13

13) Table S3 and S4 need more explanatory legends for the meaning of the columns, and for S4 how the egg specific genes were selected.

Response: We will improve the legend of Table S1–S4.

Point 14

14) Figure 6. The legend has incorrect labeling of A/B/C/D. In B what is the meaning of the scale shown?

Response: We will correct the legend as follows: "RNA-seq of the female gametophyte cells. The biological replicates were sequenced for the two SY, two CC, three EC, and four mSY cells. **(A)** Venn diagram of the expressed genes (> 10 reads) in each cell type. **(B)** Hierarchical clustering of samples for the RNA-seq. Scale bar means height. **C** The PCA analysis of all transcriptome data, the female gametophyte cells, ovules and seedlings. **D** Heatmap of the differentially expressed genes between the synergid cells in the wild type and the *myb98* mutant. OV, ovule; SL, seedling. We will improve the method in the manuscript for the hierarchical clustering analysis as follows: Hierarchical clustering analysis according to the value of the Spearman correlation between different samples was carried out using phylogram package (`https://github.com/rambaut/figtree/`).".

Point 15

15) Results 3.6 CDR1-L2 was identified as a myb98 DEG in their analysis, while SBT4.13 and EC1.1 were not, though this is not noted. Why do the authors think this is the case?

Response: In our current thresholds of DEG detection, SBT4.13 and EC1.1 were not detected as *myb98* DEGs. We confirmed the expression levels of SBT4.13 and EC1.1, which had been reported as the egg cell–specific genes. Since the expression of these genes was higher in synergid cells of *myb98* mutant than in those of wild-type, we generated a reporter lines.

Point 16

16) Line 50 - Bleckmann and Dresselhaus 2016 is referenced to indicate that SBT4.13 was known to be mis-expressed in myb98 mutant. However, this reference does not appear to contain any observations on the myb98 mutant. The authors should check this and correct the reference if needed.

Response: We made a mistake. We will correct the sentence as follows: Previously, SBT4.13 were found for the egg cell-specific gene (Bleckmann and Dresselhaus, 2016).

Point 17

17) As mentioned in the major comments, the differences in numbers between Tables 1 and 2 is unclear as they are clearly derived from the same dataset. A clear explanatory legend is needed, and they could be combined if possible.

Response: As a response above, we mistook the label of Table2. We will correct it.

Point-by-Point Response to Reviewer #2

Major comments

We thank you for your kind reviewing and helpful comments. As with your advice, we will improve our manuscript with the following points.

Point 1

1) There are a number of minor issues that should be improved (see below), but most important would be a deeper analysis of their transcriptomic data. Their data is unique, but authors did not spend much efforts to comprehensively report, for example, on genes enriched in the four cell types analyzed. Authors speculate, for example, about synergid-synergid-egg cell communication: thus it would be interesting to know whether genes encoding secreted peptide genes are preferentially expressed in the cell types or whether certain transcription factors, which are often associated with cell fate determination, are specifically expressed. Moreover, the heat map (Fig. 6D) shows DEGs, but we don't learn their identify nor anything about the biological processes that are different between the cells; e.g. do the cells vary in the cell cycle? Synergids are likely in phase G0, while eggs and central cells are supposed to be quickly activated after gamete fusion - but are they similar in this respect? Do synergid-enriched genes especially encode CRPs like in Torenia and which genes are specifically enriched in central cells? I think such bioinformatic analyses would further enhance the significance and impact of their report; these analyses are quickly done and thus, I am a bit surprised that such information is not included in the manuscript.

Response: We will perform a gene ontology analysis to characterize each cell and make some new figures.

Point 2

2) Another issue is that there is a lot of redundancy in the Discussion; results and constructs are described again, authors refer to images and figures, and partly arguments mentioned already in the Results section are picked up again - I suggest to the authors that they may consider to combine Results and Discussion

Response: We will improve the manuscript to reduce redundancy. As Reviewer #2 suggestion, we will consider the combined Results and Discussion.

Minor comments

Point 3

3) I think it is a bit strange that almost the same scheme shown in Fig. 1A is again shown in the summary figure Fig. 9; Fig. 9 is very detailed and nicely summarizes major findings of the manuscript and is slightly different to Fig. 1A, which probably aims to indicate the knowledge before this report - however, we know already more than what is shown in Fig. 1A like the formation of a second vacuole at the chalazal end of the FG at stage FG3/4. Thus, my suggestion is to remove Fig. 1A and instead draw the outlines of FG stages in Fig. 1B - I think that's sufficient as this part of the manuscript is

accompanied by a number of movies.

Response: As Reviewer #2 comment, we will delete Fig. 1A.

Point 4

4) With the exception of Figs. 5, 6 and 9 titles are missing in figure legends.

Response: We will add the titles of figures as follows:

Figure 1: Live-cell imaging of nuclear dynamics during female gametophyte development in *Arabidopsis* using an in vitro ovule culture system

Figure 2: Live-cell imaging of cellularization and maturation during FG4–FG5

Figure 3: Live-cell imaging of cell fate specifications during FG4–FG5

Figure 4: Nuclear dynamics in the synergid cells of *myb98*

Figure 7: Expression of EC–specific genes in the *myb98* synergid cells

Considering Reviewer #2 comment below, we will delete Fig 7A, move Fig 7B and 7C to Supplemental Figure, and change Fig 8 to Fig. 7.

Point 5

5) In botany the definition of the egg apparatus is "A group of three cells, consisting of the egg and two synergid cells, in the micropylar end of the embryo sac in seed plants." It does not make sense to include the central cell as part of the egg apparatus as done by the authors; the central cell is also fertilized and considered by many researchers as a second female gamete.

Response: We apologize the incorrect description. We will correct the sentence as follows: "Except for the antipodal cells, the central cell and the egg apparatus which consists of the egg cell and the synergid cells are essential for sexual reproduction.".

Point 6

6) In the description about RNA-seq in the Introduction authors constitute that rice and Arabidopsis data have been generated using LAM; this is partly not correct and authors should mention that - similar to their study - gametes from rice, maize and Arabidopsis were manually isolated after enzymatic treatment (e.g. rice: Anderson et al., 2017; maize: Chen et al., 2018; Arabidopsis: Zhao et al., 2019).

Response: We apologize for the grammatical mistake to express the cell collected by LAM is only the central cell in Arabidopsis. We will improve the sentence as follows: "In plant reproduction research, the protoplasts of gametes which were manually isolated from rice, maize, *Amborella* and *Arabidopsis* have been investigated by RNA-seq (Anderson et al., 2013; Chen et al., 2017; Flores-Tornero et al., 2019; Zhao et al., 2019). *Arabidopsis* central cells collected by LAM also were investigated by RNA-seq (Schmid et al., 2012).".

Point 7

7) I don't think authors introduced the LTI6b marker - does it label the whole plasma membrane or only locally? It appears that the membrane at the micropylar pole was not labelled.

Response: The whole plasma membrane was labeled with LTI6b–tdTomato. Depending on the line, only the plasma membrane of female gametophyte cells was labeled, not sporophytic cells, in some

RPS5Apro::tdTomato–LTI6b lines. We think the difficulty in seeing the membrane at the micropylar pole is due to the strong signal of the filiform apparatus.

Point 8

8) Authors conclude that the NLS marker was slower detectable compared to the mitochondrial marker; however, they have used different promoters: EC1.1 and EC1.2 - perhaps the genes are activated differently after cellularization - this should be discussed; I am also surprised that many figures involve EC1 promoters/genes which were described in Sprunck et al. 2012, but this paper is not mentioned.

Response:

NLS marker was also slower detected in the MYB98 promoter case, so we believe NLS marker was slow. But as Reviewer #2 says, there is a possibility that the expression of EC1.1 and EC1.2 genes is different. We will discuss about the possibility and also cite Sprunck et al. (2012) appropriately.

Point 9

9) In the text authors first mention Fig. 5B and then Fig. 5A - this should be switched, either in the text or in the figure.

Response: We will improve the sentence as follows: "We treated the ovules in emasculated ovaries of the transgenic marker line for the synergid cells, *MYB98pro::GFP*, with enzyme solutions(Figure Fig 5A, 5B). "

Point 10

10) Page numbers are missing for most references.

Response: We will add page numbers of references appropriately.

Point 11

11) Fig. 5: In the legend authors should indicate the marker lines they have used to labe the cells.

Response: We will add the marker lines to the Figure5 legend.

Point 12

12) Fig. 6B and C are redundant - C gives even more information and thus B should be removed from the manuscript.

Response: In Hierarchical clustering (Fig. 6B), we would like to show that the clade of *myb98* synergid cell is combined into the same clade as the egg cell, not the synergid cell clade. We will improve the manuscript to add the above information by referring to Fig. 6B.

Point 13

13) In Fig. 7+8 authors analyze genes highly expressed in eggs and show that expression is expanded in myb98 mutant ovules - I think they should combine the figures; the phylogram in Fig. 7A is anyhow not very informative and the information can be sufficiently described in the text.

Response: As Reviewer #2 suggestion, we will only describe the CDR1 genes in the text and arrange

Fig 7 and 8 as described above.

Point-by-Point Response to Reviewer #3

Major comments

We thank you for your kind reviewing and helpful comments. As with your advice, we will improve our manuscript with the following points and shortened introduction.

Point 1

1) Page 2- First column-line 16- How will a reader from a non-plant field understand what is the ”chalazal axis”?

Response: We will modify the sentence to understand the micropyle–chalazal axis for a non-plant field as ”the micropyle–chalazal (distal–proximal) axis”.

Point 2

2) Page 2- First Column- line 24- Please revise this sentence. Now it sounds like the pollen tube carries only one sperm cell.

Response: We will modify the sentences as follows: ”In flowering plants, two female gametes are fertilized by two sperm cells carried by the pollen tube. The egg cell is one of the female gametes that forms the embryo in the seeds by fertilization with the sperm cell. The central cell is regarded as one of the gametes because it fertilizes the sperm cell, but the fertilized central cell forms the embryo-nursing tissue endosperm in the seed, and it is not inherited in the next generation.”

Point 3

3) Page 2- Second column- line 46-47- SC and pollen VN are not protoplasts. Please correct.

Response: We will improve the sentence as follows: ”In plant reproduction research, the protoplasts of gametes which were manually isolated from rice, maize, *Amborella* and *Arabidopsis* have been investigated by RNA-seq (Anderson et al., 2013; Chen et al., 2017; Flores-Tornero et al., 2019; Zhao et al., 2019).”.

Point 4

4) Page 2- Second column- line 52- Please explain what ”greater levels” means.

Response: We will improve the sentence as follows: ”These reports have demonstrated that RNA-seq could analyze the genome-wide gene expression profiles at higher resolutions than microarrays.”.

Point 5

5) Page 2- Second column- line 54-57- This is an overstatement. We are far from knowing all those genes.

Response: As Reviewer #3 suggests, we will improve the sentence as follows: Although some genes responsible for each cells function have gradually been elucidated from these studies, the function of many other individual cell–specific genes is still unknown.

Point 6

6) Page 3- First column- First paragraph (line 1-19)- This paragraph contributes little to the while story. Consider deletion.

Response: In this study, we originally optimized the condition to isolate the female gametophyte cells in *Arabidopsis*. This paragraph indicates the motivations to optimize the enzyme solution for female gametophyte cell isolation in *Arabidopsis*. Therefore we believe this paragraph is necessary.

Point 7

7) Page 3- First column- First paragraph (line 47)- "we then built a technology platform for transcriptome analysis" - standard kits and sequencing technology were used. Consider re-phrasing.

Response: We will improve the sentence as follows: We then performed transcriptome analysis using a next-generation sequencer for a small number of isolated female gametophyte cells.

Point 8

8) Page 3- Second column - First Paragraph (line 2-12)- Different transgenic lines are described. Are they in Col background?

Response: All transgenic lines are Col background. To clarify, we modified the sentence as follows: "All *Arabidopsis thaliana* transgenic lines were in the Columbia (Col-0) background, and *myb98* mutant has been described previously (Kasahara et al., 2005)."

Point 9

9) Page 4- First column- line 24-27- How many independent transgenic plants per line were analyzed? And where can the primers be found?

Response: We will provide the information the number of transgenic lines, trials, and primers as Supplemental Tables.

Point 10

10) Page 4- First column- Section 2.2 Microscopy- Which results were acquired with which system? This should be clarified. Can be a supplemental file.

Response: We also provide the microscope information for each result as a Supplemental Table.

Point 11

11) Page 4- Second column- Section 2.3. More detailed are required, for eg: Treated for how long? This paragraph would benefit from more details.

Response: We will improve the sentence as follows: "We used an inverted fluorescence microscope (IX-71; Olympus, Japan) equipped with a three-charge-coupled device (CCD) digital camera (C7780; Hamamatsu Photonics Ltd., Japan). Images were acquired using a 40× objective lens (LUCPlanFl 40×, WD = 2.7–4 mm, NA = 0.60; Olympus). We emasculated the flowers at stage 12c (Christensen et al., 1997). Two days later, the unfertilized ovules of each cell marker line were treated with enzyme solution

(1 % cellulase [Worthington, USA], 0.3 % macerozyme R-10 [Yakult, Japan], 0.05 % pectolyase [Kyowa Kasei, Japan], and 0.45 M mannitol [pH 7.0]). To collect the target cells during 20 minutes to 1 hour of enzyme solution treatment, we used a micromanipulator (MN-4, MO-202U; Narishige, Japan) and micropipette (Picopipet HR; Nepa Gene, Japan) with glass capillaries (G-1; Narishige, Japan), which were pulled with a micropipette puller (P-97; Sutter, USA) (Ikeda et al., 2011).".

Point 12

12) Page 4- Second column - Section 2.4- Do these numbers (12-18) apply to all three cell types? RNA-seq experimental method needs to be a bit more detailed, e.g. information on sequencing depth is missing.

Response: We will add the detail in RNA-seq libraries and describe the correlation between the replicates.

Point 13

13) Page 4- Second column- section 2.5 line 39 - Why were two different analysis methods used?

Response: TCC was used to identify the DEGs between two groups. TCC+baySeq could detect the DEGs among three gropus. We used additional analysis for three group comparison to more accurately classify DEGs in each group.

Point 14

14) Page 5- First column - First paragraph (line 1-23)- This whole paragraph can be fused with the introduction.

Response: As Reviewer #3 advice, we will fuse this paragraph with the Introduction.

Point 15

15) Page 5- First column- Line 41-45- How can the authors deduce these specific movements from the videos? How do they know where the ”chalazal-micropylar axis” is, and the ”surface of the female gametophyte”? In fixed material one could probably define this, but not from the videos shown here.

Response: We can see the shape of the female gametophyte as the background signals in the over-enhanced images as follows.

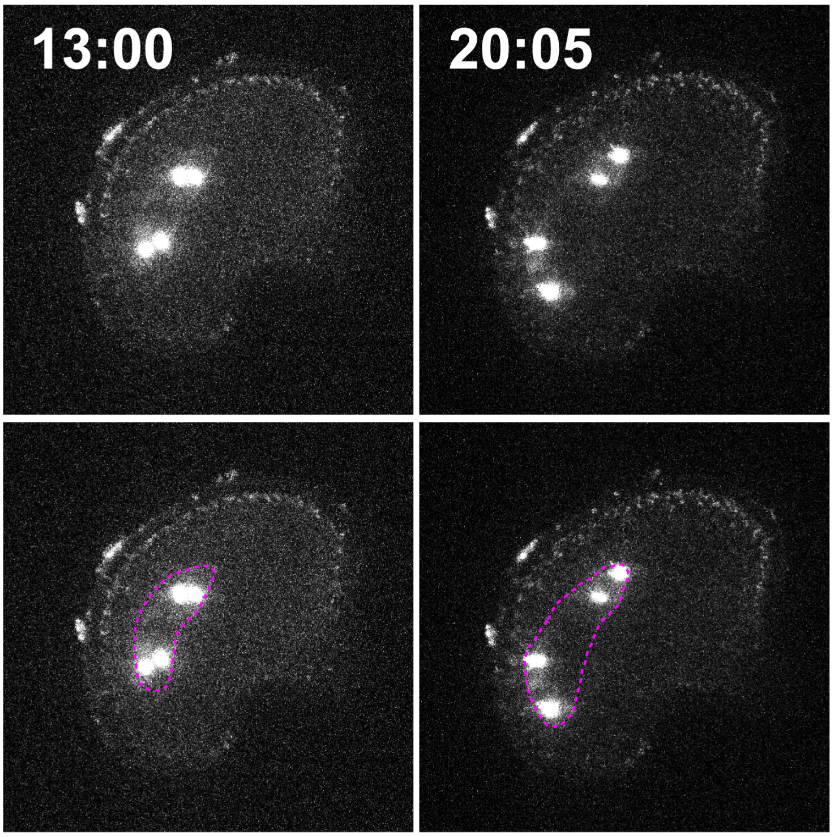

Point 16

16) Figure 1B: The authors need to provide a way to identify different stages. It is very difficult to follow the development process based on the images. One solution could be to merge Figure 1A and Figure 1B and show a cartoon with microscopic images.

Response: Considering the all Reviewers comments, we will remove Figure 1A. We identified the FG1, FG2, FG4, FG5 stages by counting the number of nuclei. FG3 was distinguished by the fact that the two nuclei begin to move to both ends. We can identify each mitosis because we can see the chromosomes, not nuclei.

Point 17

17) Page 5- First column- line 52- It would help to mention what these markers mark.

Response: We will make the list of each construct as a supplemental table.

Point 18

18) Page 5- First column - line 53- N=1 is insufficient.

Response: We plan to add the data of RPS5Apro::H2B-sGFP, so we are growing the seeds now. We will need two more months to observe and analyze them.

Point 19

19) Page 6- First column- line 29- Have the authors considered that the promoter sequence might not be complete and regulatory sequences are missing in this construct? Expression of MYB98 might also be regulated by regulatory regions in introns of the genes or in the UTR (both missing in this construct?). What about in situ hybridization? Or are there any transcriptome data for megagametogenesis for this stage that would corroborate these observations? Another important question would be in how many independent transgenic lines this was observed - MYB98pro might drive GFP expression here due to an effect of the integration site into the genome.

Response: This MYB98pro::GFP line was established by Kasahara et al. (2005), and there is only one line. This promoter sequence consists of 1674 bp of sequence upstream of the translational start codon and 72 bp of the first exon of MYB98. We have no data on in situ hybridization and transcriptome data for megagametogenesis. We plan to observe the protein fusion construct (MYB98pro::MYB98-GFP; (Punwani et al., 2007)) and will need two more months to grow and observe the plants.

Point 20

20) Page 6- First column- line 36- I do not see this increase. If anything, a decrease in synergid cells. If this claim is to stand, measurements of GFP might be needed.

Response: We will measure the fluorescent signals of MYB98pro::GFP and MYB98pro::MYB98-GFP.

Point 21

21) Page 6- First column- line 41- The authors use RPS5Apro::tdTomato-LTI6b construct. Why? Some explanation would help here.

Response: Depending on the line, only the plasma membrane of female gametophyte cells was labeled, not sporophytic cells, in some RPS5Apro::tdTomato–LTI6b lines. At first, we used this promoter to construct the marker line for female gametophyte imaging. However, we recently found that the GPR1 promoter can label only the female gametophyte cells, so we made a nuclear marker line, but we haven't made a plasma membrane marker line yet.

Point 22

22) Page 6- Second column- line 30- The authors claim "the expression of NLS-mRuby2 was slower than that of the free GFP". It can also be possible that the insertion points into the genome have an influence on expression timing. How many independent transgenic lines were analysed? Page 6- Second column- line 46- There seems to be a control missing here: Measurements in the line MYB98pro::GFP, MYB98pro::NLS-mRuby2 in wild type. Does this one behave as wild type (as one would expect) or like what is observed in myb98 mutant? In the latter case expression of MYB98pro::GFP would cause the

observed phenotype.

Response:

Unfortunately, we have not MYB98pro::GFP, MYB98pro::NLS–mRuby2 in Col. So, we will analyze additional independent lines of MYB98pro::GFP, MYB98pro::NLS–mRuby2 in *myb98* and will need two more months to grow and observe the plants. And now, we try to transform the MYB98pro::GFP, MYB98pro::NLS–mRuby2 in Col., but three months are needed for analysis of T1 plants.

Point 23

23) Page 7- First column- line 23- Why are the synergid cells so large in this image? And the size of the scale bar is missing in the legend.

Response: Scale bar indicate 20 μm. The size of the ovules depends on the size of the plant body. The diameter of the synergid cell protoplasts are about 10 to 20 micrometers.

Point 24

24) Page 7- Second column-line 3 - The authors should consider introducing the result of DGE here, instead of mentioning it only in the discussion.

Response: As Reviewer #3 advice, we will improve this paragraph describing about DEGs.

Point 25

25) Page 7- Second column-line 1 and 2- The results of the PCA do not support the claim that ”The expression profile of the synergid cells in the mutant was more like that of the egg cells than the synergid cells in the wild type”. This needs to be explained better.

Response: In Hierarchical clustering (Fig. 6B), we would like to show that the clade of *myb98* synergid cell is combined into the same clade as the egg cell, not the synergid cell clade. we will improve this sentence by referring to Fig.6B.

Point 26

26) Page 7- First column-line 51-52- The authors used read count (>10) to detect expressed genes, which is problematic. Since no sequencing depth is given, it is hard for a reader to follow. Normalised values like CPM, TPM or FPKM would be better.

Response: As Reviewer #3 comment in point12, We will add the detail in RNA-seq libraries and describe the correlation between the replicates. We will improve the data using TPM >1.

Point 27

27) Page 9- First Column- line 45- The authors write ”A previous microarray analysis of myb98 presented different results,” but do not mention what were those previous results and how were they different from what they see in this study. Also, the supplemental file with all tables needs to be arranged properly as it is too difficult to navigate and look for specific DGE results.

Response:

We will describe the differences as follows: ”Although a previous microarray analysis could not detect the change to the egg cell-like in the synergid cells of *myb98*, this is thought to be because their sample

contained the entire ovule, not the synergid cells alone (Jones-Rhoades et al., 2007).". As Reviewer #3 advice, we will add some excel sheets for each cell's DEGs.

Point 28

28) Page 9- Second column- line 33- The authors mention that "This result indicated that the synergid cells with the egg cell-specific genes were not functional for fertilization in the myb98". Is it possible that mutant pollen was affected?

Response: If the pollen tube of *myb98* is affected, fertilization with the egg cell as well as the synergid cells with the egg cell-specific genes should be affected. However, the embryo was normally observed in *myb98* mutant, so the effect of *myb98* pollen tube is unlikely.

Point 29

29) Page 9- Second column-line 30-33- Can the authors please point to the specific results they are referring here?

Response: We will add the following figure about the embryo of *myb98* homozygous mutant as a supplemental figure.

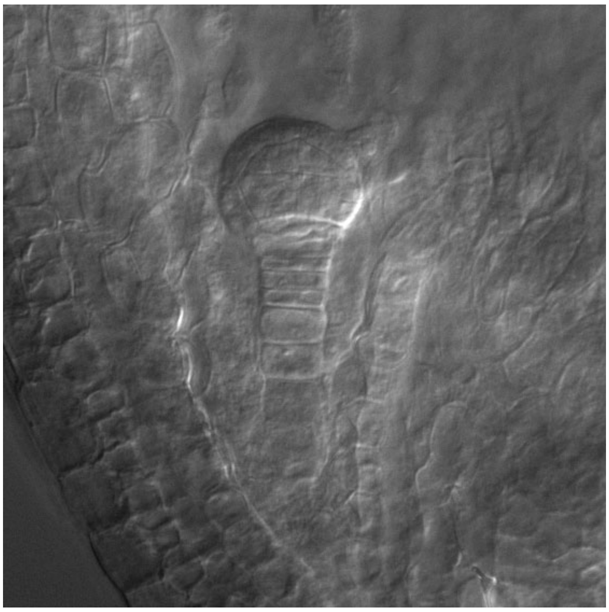

Point 30

30) Page 10- First column- The first paragraph would benefit from extensive re-writing to clarify what the authors want to convey here. The discussion on nuclei positioning and its role in initiation of synergid cell fate needs to be explained properly. Also, the role of AGL80 seems to be misplaced in this part of the discussion. The last line (line 23-25) of this paragraph reading "These findings suggested that the MYB98 may also play a role in preventing the acquisition of egg cell fate in the accessory cells" is not clear at all and needs re-writing to be coherent with the first part of this paragraph.

Response: After obtaining the results of MYB98pro::MYB98-GFP, we will modify the paragraph to understand better.

Point 31

31) Page 10- First column- line 49. The authors write ”that the synergid cells detect the abnormal conditions of the egg cell”. Please explain better. Additionally, as authors mention in several parts of results and discussion, synergids in myb98 mutants behave like egg cell with high expression of egg cell specific genes, such as CDR1L2. What is missing is the information on expression of synergid specific gene markers and how those are affected, e.g. AtLURE1.

Response: Considering Reviewer #3 comment, we will modify the sentence as follows: ”Lateral inhibition from the egg cell restricts the egg cell fate in accessory cells (Völz et al., 2013)). Based on these findings, we speculate that egg-cell failure induce a decrease of *MYB98* expression. The combination of these cell–cell communications and the flexible fate maintenance might allow for only one of the two synergid cells to become an egg cell.”

We also add the data and the references about the synergid-specific genes in *myb98* as follows: ”The expression of AtLURE1 was decreased in the *myb98* synergids (Takeuchi and Higashiyama, 2012)”

Minor comments

Point 1

1) Page 1- Second column-line 6- It remains unclear what the authors want to convey in the second part of this sentence.

Response: To better understanding, we will modify the sentence as follows: ”The development of the female gametophyte in angiosperms is an attractive model for studying cell fate specifications.”

Point 2

2) Page 5- Second column- line 43 - Supplementary Movie 3, ”later”. Is it not possible to say exactly when?

Response: To better understanding, we will change to ”second half of Supplementary Movie 3”.

Point 3

3) Page 6- First column- line 16- what does ”ovules of synergid cells” mean?

Response: We will modified the sentence as follows: ”We observed the egg cell marker, EC1.2pro::mtKaede (Hamamura et al., 2011), and the synergid cell marker, MYB98pro::GFP (Kasahara et al., 2005) (Figure 3A,B, Supplementary Movie 5, 6)”.

Point 4

4) Page 6- Second column- line 5- Avoid using time points like 1.7 h for example. Please be consistent with hour and time notation. Preferably use 1 h 15 min or 80 min.

Response: According Reviewer #3 suggestion, we will modify time notation as hr:min.

Point 5

5) Page 7- First column- line 40- What were the optimized conditions for these cell types?

Response: Egg cells were detached from ovules same as synergid cells. The frequency of released central cells were lower than that of egg cells. After 30 minutes of treatment with the enzyme solution, ovules without released central cells were gently pressed by glass capillary to help release from the ovules. We will improve the manuscript with these information.

Point 6

6) Page 7- First column- line 54 Figure 6B- Why are the numbers of replicates for samples prepared for this study different?

Response: Collection of synergid cells and central cells was much more difficult than that of egg cells and synergid cells of *myb98* mutant.

Point 7

7) Page 8- Second column-line 21- What does placenta mean and what additional conditions were determined?

Response: Considering the recent male meiosis imaging, we will modify the sentence as follows: "To observe meiosis, megasporogenesis, and other early processes *in vitro* in real-time, it was considered that further improvements were required such as male meiosis (Prusicki et al., 2019; Valuchova et al., 2020)".

Point 8

8) Page 9- Second column- line 29- "What each gene senses"? It is not clear what the authors want to say here.

Response: To better understanding, we will modify the sentence as follows: "These results suggested that the regulations of each egg-cell specific gene expression are different."

Point 9

9) Figure Legends should be thoroughly checked. For example: Figure 1C, Figure 6A-C. The contrast of letters within figure needs to be enhanced (For example: Fig. 2C where EC in blue is almost invisible). Similarly, Figure 9, yellow arrows are invisible.

Response: We will double-check the figure legend and modify the color to better visibility.

Point 10

10) Although the authors mention that English editing services were used, the manuscript needs to be improved significantly as far as English is concerned.

Response: English editing service will check again with Reviewer #3 comments in the revised manuscript.

References

Anderson, S. N., Johnson, C. S., Jones, D. S., Conrad, L. J., Gou, X., Russell, S. D., and Sundaresan, V. (2013). Transcriptomes of isolated Oryza sativa gametes characterized by deep sequencing: evidence for distinct sex-dependent chromatin and epigenetic states before fertilization. *Plant J*, 76(5):729–741.

Chen, J., Strieder, N., Krohn, N. G., Cyprys, P., Sprunck, S., Engelmann, J. C., and Dresselhaus, T. (2017). Zygotic genome activation occurs shortly after fertilization in maize. *Plant Cell*, 29(9):2106–2125.

Christensen, C. A., King, E. J., Jordan, J. R., and Drews, G. N. (1997). Megagametogenesis in Arabidopsis wild type and the Gf mutant. *Sex Plant Reprod*, 10(1):49–64.

Flores-Tornero, M., Proost, S., Mutwil, M., Scutt, C. P., Dresselhaus, T., and Sprunck, S. (2019). Transcriptomics of manually isolated Amborella trichopoda egg apparatus cells. *Plant Reproduction*, 32(1):15–27.

Hamamura, Y., Saito, C., Awai, C., Kurihara, D., Miyawaki, A., Nakagawa, T., Kanaoka, M. M., Sasaki, N., Nakano, A., Berger, F., and Higashiyama, T. (2011). Live-cell imaging reveals the dynamics of two sperm cells during double fertilization in Arabidopsis thaliana. *Curr Biol*, 21(6):497–502.

Ikeda, Y., Kinoshita, Y., Susaki, D., Ikeda, Y., Iwano, M., Takayama, S., Higashiyama, T., Kakutani, T., and Kinoshita, T. (2011). HMG Domain containing SSRP1 is required for DNA demethylation and genomic imprinting in arabidopsis. *Dev Cell*, 21(3):589–596.

Jones-Rhoades, M. W., Borevitz, J. O., and Preuss, D. (2007). Genome-Wide Expression Profiling of the Arabidopsis Female Gametophyte Identifies Families of Small, Secreted Proteins. *PLoS Genet*, 3(10):e171.

Kasahara, R. D., Portereiko, M. F., Sandaklie-Nikolova, L., Rabiger, D. S., and Drews, G. N. (2005). MYB98 Is Required for Pollen Tube Guidance and Synergid Cell Differentiation in Arabidopsis. *Plant Cell*, 17(11):2981–2992.

Köszegi, D., Johnston, A. J., Rutten, T., Czihal, A., Altschmied, L., Kumlehn, J., Wüst, S. E. J., Kirioukhova, O., Gheyselinck, J., Grossniklaus, U., and Bäumlein, H. (2011). Members of the RKD transcription factor family induce an egg cell-like gene expression program. *Plant Journal*, 67(2):280–291.

Prusicki, M. A., Keizer, E. M., van Rosmalen, R. P., Komaki, S., Seifert, F., Müller, K., Wijnker, E., Fleck, C., and Schnittger, A. (2019). Live cell imaging of meiosis in Arabidopsis thaliana. *eLife*, 8:1–31.

Punwani, J. A., Rabiger, D. S., and Drews, G. N. (2007). MYB98 Positively Regulates a Battery of Synergid-Expressed Genes Encoding Filiform Apparatus–Localized Proteins. *Plant Cell*, 19(8):2557–2568.

Schmid, M. W., Schmidt, A., Klostermeier, U. C., Barann, M., Rosenstiel, P., and Grossniklaus, U. (2012). A Powerful Method for Transcriptional Profiling of Specific Cell Types in Eukaryotes: Laser-Assisted Microdissection and RNA Sequencing. *PLoS ONE*, 7(1):e29685.

Sprunck, S., Rademacher, S., Vogler, F., Gheyselinck, J., Grossniklaus, U., and Dresselhaus, T. (2012). Egg Cell-Secreted EC1 Triggers Sperm Cell Activation During Double Fertilization. *Science*, 338(6110):1093–1097.

Susaki, D., Takeuchi, H., Tsutsui, H., Kurihara, D., and Higashiyama, T. (2015). Live imaging and laser disruption reveal the dynamics and cell-cell communication during torenia fournieri female gametophyte development. *Plant Cell Physiol*, 56(5):1031–1041.

Takeuchi, H. and Higashiyama, T. (2012). A Species-Specific Cluster of Defensin-Like Genes Encodes Diffusible Pollen Tube Attractants in Arabidopsis. *PLoS Biol*, 10(12):e1001449.

Valuchova, S., Mikulkova, P., Pecinkova, J., Klimova, J., Krumnikl, M., Bainar, P., Heckmann, S., Tomancak, P., and Riha, K. (2020). Imaging plant germline differentiation within Arabidopsis flowers by light sheet microscopy. *eLife*, 9:9–11.

Völz, R., Heydlauff, J., Ripper, D., von Lyncker, L., and Groß-Hardt, R. (2013). Ethylene Signaling Is Required for Synergid Degeneration and the Establishment of a Pollen Tube Block. *Dev Cell*, 25(3):310–316.

Wang, D., Zhang, C., Hearn, D. J., Kang, I.-H., Punwani, J. A., Skaggs, M. I., Drews, G. N., Schumaker, K. S., and Yadegari, R. (2010). Identification of transcription-factor genes expressed in the Arabidopsis female gametophyte. *BMC Plant Biol*, 10(1):110.

Zhao, P., Zhou, X., Shen, K., Liu, Z., Cheng, T., Liu, D., Cheng, Y., Peng, X., and Sun, M.-x. (2019). Two-Step Maternal-to-Zygotic Transition with Two-Phase Parental Genome Contributions. *Dev Cell*, 49(6):882–893.e5.
