# Supplementary material for: Dynamics of the cell fate specifications during female gametophyte development in Arabidopsis
Source: PLoS Biol. 2021 Mar 26;19(3):e3001123. doi: 10.1371/journal.pbio.3001123 (PMC7997040; doi:10.1371/journal.pbio.3001123)
Supplement: Attachment — Submitted filename: Review_Commons_Reviewers_comments.pdf [file pbio.3001123.s038.pdf]

Email

**Manuscript #**	RC-2020-00249
**Title**	Dynamics of the cell fate specifications during female gametophyte development in Arabidopsis
**Corresponding Author**	Dr. Daisuke Kurihara (Nagoya University)
**Date:**	2020-06-10 05:51:25
**Last Sent:**	2020-06-10 05:51:25
**Created By:**	Redacted
**From:**	s.monaco@reviewcommons.org
**To:**	kuri@bio.nagoya-u.ac.jp
**Subject:**	Review Commons Reviews for Refereed Preprint RC-2020-00249
**Email**	June 10, 2020

RE: Review Commons Refereed Preprint #RC-2020-00249

Dr. Daisuke Kurihara
Nagoya University
Institute of Transformative Bio-Molecules (ITbM)
Nagoya, Aichi 464-8601
Japan

Dear Dr. Kurihara,

Thank you for sending your manuscript entitled "Dynamics of the cell fate specifications during female gametophyte development in Arabidopsis" to Review Commons. We have now completed the peer review of the manuscript. Please find the full set of reports below.

To access your manuscript online and proceed to the next steps, please use the link below:

Link Not Available

NEXT STEPS:

1. RESPONSE TO REVIEWERS: You have *four weeks* to upload to the system an *initial* response/revision plan to your reviewed manuscript (please use the link above). If you need more time to draft this document, please let us know. At this stage, a full revision is NOT expected! You have nevertheless the option to upload a preliminary revised version of the manuscript (e.g. to fix minor points or textual changes) if you prefer to do so before forwarding your manuscript to one of our affiliated journals; please ensure that all changes are visibly tracked in the file.

2. POSTING THE REVIEWS ON BIORXIV: Once the response letter has been uploaded, you will be able to instruct Review Commons to post the reviews and your response on bioRxiv alongside your preprint. Please follow the instructions that are provided online on the submission screen.

3. TRANSFER TO AFFILIATE JOURNALS: When you submit the response/initial revision plan (see also https://www.reviewcommons.org/authors/#initial-revision), you will be presented with a screen that allows you selecting the affiliate journal to which you want to submit. Please note that the full

set of reviews will be transferred alongside your response and the original manuscript. If you elected to upload an initial revised manuscript, it will be transferred alongside the original version.

4. EVALUATION AT AFFILIATES: Upon transfer, the recipient journal will consider the manuscript as a formal submission and use the transferred reviews and your response/revision plan to make an initial decision. Note that while it is under consideration at an affiliate journal, the manuscript cannot be transferred to any other affiliates.

5. NEW ROUND OF TRANSFER: If the first journal declines, you will be directly informed by this journal. The manuscript will then be unlocked on the Review Commons site so that you can initiate up to two additional rounds of transfer.

Please consult our detailed Guidelines for further details on the process and policies of Review Commons at http://reviewcommons.org/authors . If you have any further questions, please contact me directly.

Thank you again for sending us your work. We hope you will find the reviews useful and the Review Commons helps to disseminate your peer-reviewed research faster and more efficiently.

Best wishes,

Sara Monaco
--
Sara Monaco, PhD
Managing Editor
Review Commons

Reviewer #1 (Evidence, reproducibility and clarity (Required)):

This study presents live imaging of the developing female gametophyte in Arabidopsis. They use the in vitro ovule culture method which they previously established for studying early embryogenesis in Arabidopsis. Starting with the haploid functional megaspore formed after meiosis and ending with the fusion of the polar nuclei that constitute the central cell, the study presents a detailed sequence of nuclear divisions and movement, timing and location of plasma membrane formation, and the timing of expression of genetic markers associated with specific cell types. In addition, the authors present an improved method for isolation of specific gametophytic cells from the ovule and describe results from transcript profiling of these cells. The role of the transcription factor MYB98 in development of synergid cells, accessories to the egg cell that are important for fertilization, is re-assessed using these techniques, through live imaging of developing myb98 mutant gametophytes with cell-type markers, and transcriptomic analysis of myb98 mutant synergids.
The authors use their study to define and fill-in gaps in the known development of the Arabidopsis embryo sac, such as routes of migration of the polar nuclei. The authors are interested in understanding the role of nuclear position in cell-fate determination and maintenance. They seek to address the timing of cell fate acquisition, by tracking the activity of cell-type specific markers prior to or after cellularization and observing nuclear dynamics. They find that cell-fate specification initiates at the time of cellularization, starting with the egg cell and quickly followed by other cell types. The authors show that myb98 synergids acquire partial egg-like characteristics but continue to express MYB98. This implies that other factors are required for initiation and regulation of synergid identity and the authors propose nuclear position as the regulating factor. In the case of the egg cell and egg-like mutant synergids, observed differences in the timing of expression of various egg cell genes are interpreted to mean that complex regulation is required to acquire and

maintain cell-specific properties. Finally, they note that synergid fate change often occurs to only one synergid, see this as evidence of synergid-to-synergid communication and suggest this is an evolutionary mechanism to ensure fertilization.

Major comments

The use of the ovule culture system and live imaging represent a significant technological advance for developmental studies of the female gametophyte. The results described in this paper are overall interesting and the experiments are technically excellent. The live imaging analysis generally agreed with developmental stages described previously, but added interesting new details about nuclear positioning and migration, division planes and the dynamics of cellularization in the gametophyte.

Some conclusions need better support from the data or qualification as detailed below:

1.In wild type they note early detection of MYB98pro::GFP throughout the gametophyte prior to cellularization and rapid activation of the EC genes in the newly formed egg cell. However, an important caveat is in the pre-cellularized state (the coenocytic gametophyte), the movement of free GFP, and that of NLS-mRuby2 prior to nuclear import, are both likely and possible respectively. Thus, marker expression will not necessarily mirror localization of MYB98 protein prior to cellularization. Moreover, the longevity of the relatively stable fluorescent proteins could lead to the appearance of expression in newly formed egg and central cells.

2.They find that the position of nuclei in the synergids after cellularization is not maintained in myb98 mutants and nuclear positioning becomes more like that in the egg cell. This leads to a muddled message about the role of nuclear position leading to cell fate (and gene expression) vs. gene expression leading to nuclear position: Nuclear position regulates MYB98 initiation but, in myb98, nuclei move around; later MYB98 is still expressed despite this nuclear movement. This apparent contradiction should be more clearly discussed and possible explanations provided.

3.The role of MYB98 in cell fate is investigated by comparing wild type and mutant synergid transcriptomes and cell type marker expression. The authors show that some egg-specific genes are expressed in synergids using transgenes. They conclude from their transcriptomic analysis that mutant synergids are more similar to egg cells.

a. Their RNAseq analyses are not sufficiently well described for full understanding of the results, as detailed in minor comments below. Importantly for low-input RNA libraries, the authors should describe the correlation between replicates statistically, and strategies for overcoming amplification error.

b. It appears that their experiments showed both egg and central cells genes are expressed in mutant synergids. From Figure 6A: 225 egg specific genes are found in the myb98 synergid (22% of egg specific genes). However, there are an even greater number of central cell genes mis-expressed in mutant synergids: 838 central cell genes that are turned on (15% of central cell specific genes). Even when only DEGs (SY vs mSY) are used (Figure 6D) and from looking at Table S3 (21 egg genes vs 17 CC genes), there are clearly a comparable number of CC genes expressed in mutant synergids. These central cell expressed genes are not sufficiently addressed in the text. The authors choose to test only egg cell genes by promoter-reporter constructs. The somewhat closer clustering of mutant synergids with egg cells is an indication that overall the cells are more egg cell-like but if that is the most important piece of evidence there should be more details about how the analysis was done. Given the other points above, other interpretations should be considered.

c. There is little discussion of the actual genes that are changed in expression in the myb98 mutant. Do these provide any evidence of acquisition of cell fate, such as increased expression of egg-cell

transcription factors (RKD family) or other regulation?

4. The authors state that the maintenance, not initiation, of synergid genes was defective in myb98. However, they only give early expression information for MYB98, and note that the synergids were phenotypically abnormal prior to maturity. No other synergid genes were examined to observe their expression dynamics. No discussion is made of synergid specific genes that were unaffected in the RNAseq of mutants. Some egg genes were observed just after cellularization, indicating an early change from synergid to egg-like. The conclusion that synergids are initiated correctly do not appear to be supported by the data presented.

5.MYB98pro::reporter signals were observed throughout the gametophyte prior to and just after cellularization. The expression of one of three examined egg-specific genes (SBT4.13) was observed in antipodals 30% of the time. In contrast, two other genes were observed only in egg and synergid cells. From this, the authors suggest MYB98 negatively regulates egg cell fate in both synergids and antipodals.

a. As mentioned in the first comment, the authors do not account for the movement and persistence of fluorescent reporter proteins which means the true presence of MYB98 protein in antipodals is uncertain. A translational MYB98-GFP fusion should be used if the authors wish to conclude function from reporter expression.

b. The number of examined ovules in Tables 1 and 2 (from 6 to 23 ovules) is much lower than typically used to determine trends in gene expression. If these are live imaging experiments then from each experiment, there might be multiple observations of the same ovule, which provides more confidence than a single observation, and might be the reason for the lower number observed. However, to be convincing on the specific points regarding gene expression in the mature FG6 gametophyte in the WT and mutant ovules, a more conventional analysis with greater numbers of observed ovules (n>50) is recommended.

6.The authors state that there is usually conversion of only one of the synergids to an egg-like state and that this is similar to the known synergid-to-egg conversion when the egg is defective. The authors conclude that this indicates synergid-to-synergid communication. The evidence shown does not make clear that this is the case.

a. For CDR1-L2 and SBT4.13 markers, figures 7C and 8B do not show single synergids, and the information in Table 2 is insufficiently explained to be understood. If these are the same observations as described in Table 1 - what makes the two Tables different is quite unclear (aside from the obviously different numbers in each sub-category). If single synergids are being counted, then shouldn't the total should be double?
b. It is not stated how many times the EC1.1pro expression (Fig8C) was observed.

c. Finally, even if this were clear, a simpler explanation would be that once the synergid acquires some egg-like characteristics, this may include the ability to signal to neighboring synergids to prevent another egg from developing as has been described for egg cells. In other words, what is being observed is another manifestation of the previously described egg cell to synergid communication, rather than synergid to synergid communication.

Minor comments

1.All figures and Supplemental movies It is important for each time-lapse experiment to clearly state in the legend or text the number of cells observed for each construct.

2.Results 3.1 Figure 1A - the model of nuclear movement does not match that described in the text, which is shown in Figure 9. This is confusing to the reader. Figure 1A could be replaced with a

simpler version that only shows the stages and not nuclear movements or planes of division. The legend has C mislabeled as B.

3.It would useful for the authors to compare the findings from Arabidopsis with their prior results on nuclear movement from Torenia (Susaki et al, 2015).

4.Results 3.3 and beyond There is a bewildering array of promoters and reporters which make the manuscript very difficult to follow, especially as some of the promoters are from genes that are somewhat obscure. It is highly recommended that the authors provide a table to show different promoters, markers, cellular localization and their previously known stages of detection.

5.Line 15/16 "in the ovules of the egg cells and the MYB98pro::GFP ovules of the synergid cells" This sentence needs editing, as the meaning is not clear.

6.Line 24 ABI4 needs reference

7.Figure 3B The black/white pictures of Myb98::GFP are somewhat fuzzy, and definitely not as clear as the colored images with the other marker.

8.Figure 3E and Movie S8 There is a missing marker not noted on the figure and movie (purple sporophytic, not AKV), and AKV not labeled in Figure. The use of the non-contrasting sporophytic background marker makes this experiment hard to see clearly.

9.Results 3.4 Please indicate the number of observations made. Figure 4 should be all in color. If equivalent pictures of MYB98pro::GFP/NLS-Ruby in WT are available these should be used, as it is difficult to see the extent of the synergid in A.

10.Line 50 states that the myb98 synergids were more elongated but it is not possible to see this from the figure, nor are any other measurements given.

11.Results 3.5
Figure S1A. A bright field image is needed to show that there are no attached cell debris or other cells.

12.Figure S1 B legend reads: "F test of frequency showed that there is no significant difference between the two variances ($p > 0.05$)." However, the figure has a single asterisk between the two columns which should indicate that there was a significant difference.
RNAseq: With such few cells (12-18) it is important to show that reps are consistent, which was partially done using the PCA and clustering. However, more details are needed, including a correlation analysis. Did the authors use methods to reduce PCR duplicates which would be common from low-input libraries? How did the datasets compare with other cell-specific datasets (Schmid, 2012 and Zhou, 2019). Was there evidence of contamination between cell-types by looking at TPM of genes with known expression patterns?

13.Table S3 and S4 need more explanatory legends for the meaning of the columns, and for S4 how the egg specific genes were selected.

14.Figure 6. The legend has incorrect labeling of A/B/C/D. In B what is the meaning of the scale shown?

15.Results 3.6 CDR1-L2 was identified as a myb98 DEG in their analysis, while SBT4.13 and EC1.1 were not, though this is not noted. Why do the authors think this is the case?

16.Line 50 - Bleckmann and Dresselhaus 2016 is referenced to indicate that SBT4.13 was known to be mis-expressed in myb98 mutant. However, this reference does not appear to contain any

observations on the myb98 mutant. The authors should check this and correct the reference if needed.

17.As mentioned in the major comments, the differences in numbers between Tables 1 and 2 is unclear as they are clearly derived from the same dataset. A clear explanatory legend is needed, and they could be combined if possible.

Reviewer #1 (Significance (Required)):

This work presents a significant technical advance for live imaging of the Arabidopsis female gametophyte, allowing tracking of nuclei, plasma membrane formation and cell-fate via markers. The set of videos provided is truly impressive for both spatial and temporal resolution, and helps to clarify many of the missing details in the transitions between the different stages of embryo sac development. Although this method was previously applied to Torenia female gametophyte by the same authors, the conclusions here are more detailed, and are supported by the far greater availability of multiple cell-type markers and reporters using Arabidopsis (Susaki et al. 2015). As such, the method and the fine details of female gametophyte development revealed by this study are a valuable addition to the field of plant reproductive biology. The transcriptomic experiment represents the first description of an isolated mutant female gametophyte cell-type and the full dataset is very useful to the research community due to the difficulty in creating these sets. The analysis of the myb98 mutant by several methods adds knowledge about the role of this important regulator, as well as confirming the importance of positional information for differentiation.

In terms of novel biological insights, the immediate impacts are somewhat more limited. Through no fault of the authors, the findings on wild-type embryo sacs refine, but do not significantly change the existing conceptual thinking in the field. As an example of the latter, detailed imaging studies of double-fertilization by some of the same authors revealed the fusion of the surviving synergid with the fertilized central cell (Maruyama et al 2015) - a quite unexpected finding with significant implications for understanding the control of fertilization. Manipulations by laser ablation, previously accomplished in their Arabidopsis embryogenesis study, might have provided new insights, but possibly that was too technically difficult to attempt with female gametophytes. What they have done is to use a genetic mutation instead, i.e, myb98, but their conclusions from their mutant studies generally reinforce or supplement what was previously known about this gene. It was already shown to be a transcription factor that is required for synergid cell function but not specification, and putative target genes downstream of MYB98 have been previously identified (Punwani et al. 2008). In this respect, although their method for isolation of synergid cells is novel, transcriptomes of individual cell types (including synergid cells) have been previously described in Arabidopsis using other methods (e.g. Wuest et al. 2010). As detailed in the major comments, the conclusions from the transcriptomic analysis are not clear cut, and in their present manuscript, they do not reveal a new or unexpected mechanism in female gametophyte development or synergid cell differentiation. This is not a criticism of their technical achievements, its just that these achievements have not in this specific study led to corresponding leaps in understanding.

The audience interested in this work would include plant developmental biologists, and especially plant biologists studying fertilization.

The reviewer's field of expertise is the molecular biology and genetics of flowering plant reproduction, including aspects of gametophyte and seed development.

Reviewer #2 (Evidence, reproducibility and clarity (Required)):

SUMMARY:

Susaki et al. report here about female gametophyte (FG) development and cell specification using the model plant Arabidopsis. By using advanced live-cell imaging of a huge set of markers generated by the authors and an improved in vitro culture method, authors were able to visualize the dynamics and determine the timing of FG development from stages FG1 to FG6. Until now, FG development was only analyzed using fixed material and a precise timing was not known. Authors show nuclei division and division planes, nuclei migration, indicate vacuoles formation, establishment of cellularization, polar nuclei migration and fusion, cell elongation and establishment of cell fates. These finding are therefore not only of interest for plant researchers working on FG development and fertilization, authors also address fundamental processes and questions in general cell biology.

The description of FG development is accompanied by 15 unique movies, which represent a strength of the report. Thus, this part of the manuscript also provides a solid fundament for many future studies involving, for example, various mutants, analyses of additional markers studying the involvement of the cytoskeleton etc. Additionally, authors describe in the second part of their manuscript the isolation of egg and central cells as well as synergids from the mature FG and generate RNA-seq data. Remarkable are the finding that cell fates are specified almost immediately after cellularization at the eight-nucleate stage at FG5 and that synergids of the myb98 mutant display a transcriptome more similar to that of the egg cell. This finding is confirmed by the analysis of egg cell-specific genes that show a broadened expression pattern in myb98 mutant ovules.

MAJOR COMMENTS:

Overall, the key conclusions are convincing, and the methods are well presented. I am impressed about the statistical data as hundreds of movies have been recorded and analyzed.

1.) There are a number of minor issues that should be improved (see below), but most important would be a deeper analysis of their transcriptomic data. Their data is unique, but authors did not spend much efforts to comprehensively report, for example, on genes enriched in the four cell types analyzed. Authors speculate, for example, about synergid-synergid-egg cell communication: thus it would be interesting to know whether genes encoding secreted peptide genes are preferentially expressed in the cell types or whether certain transcription factors, which are often associated with cell fate determination, are specifically expressed. Moreover, the heat map (Fig. 6D) shows DEGs, but we don't learn their identify nor anything about the biological processes that are different between the cells; e.g. do the cells vary in the cell cycle? Synergids are likely in phase G0, while eggs and central cells are supposed to be quickly activated after gamete fusion - but are they similar in this respect? Do synergid-enriched genes especially encode CRPs like in Torenia and which genes are specifically enriched in central cells? I think such bioinformatic analyses would further enhance the significance and impact of their report; these analyses are quickly done and thus, I am a bit surprised that such information is not included in the manuscript.

2.) Another issue is that there is a lot of redundancy in the Discussion; results and constructs are described again, authors refer to images and figures, and partly arguments mentioned already in the Results section are picked up again - I suggest to the authors that they may consider to combine Results and Discussion

MINOR COMMENTS

3.) I think it is a bit strange that almost the same scheme shown in Fig. 1A is again shown in the summary figure Fig. 9; Fig. 9 is very detailed and nicely summarizes major findings of the manuscript and is slightly different to Fig. 1A, which probably aims to indicate the knowledge before this report - however, we know already more than what is shown in Fig. 1A like the formation of a second vacuole at the chalazal end of the FG at stage FG3/4. Thus, my suggestion is to remove

Fig. 1A and instead draw the outlines of FG stages in Fig. 1B - I think that's sufficient as this part of the manuscript is accompanied by a number of movies.

4.) With the exception of Figs. 5, 6 and 9 titles are missing in figure legends.

5.) In botany the definition of the egg apparatus is "A group of three cells, consisting of the egg and two synergid cells, in the micropylar end of the embryo sac in seed plants." It does not make sense to include the central cell as part of the egg apparatus as done by the authors; the central cell is also fertilized and considered by many researchers as a second female gamete.

6.) In the description about RNA-seq in the Introduction authors constitute that rice and Arabidopsis data have been generated using LAM; this is partly not correct and authors should mention that - similar to their study - gametes from rice, maize and Arabidopsis were manually isolated after enzymatic treatment (e.g. rice: Anderson et al., 2017; maize: Chen et al., 2018; Arabidopsis: Zhao et al., 2019).

7.) I don't think authors introduced the LTI6b marker - does it label the whole plasma membrane or only locally? It appears that the membrane at the micropylar pole was not labelled.

8.) Authors conclude that the NLS marker was slower detectable compared to the mitochondrial marker; however, they have used different promoters: EC1.1 and EC1.2 - perhaps the genes are activated differently after cellularization - this should be discussed; I am also surprised that many figures involve EC1 promoters/genes which were described in Sprunck et al. 2012, but this paper is not mentioned.

9.) In the text authors first mention Fig. 5B and then Fig. 5A - this should be switched, either in the text or in the figure.

10.) Page numbers are missing for most references.

11.) Fig. 5: In the legend authors should indicate the marker lines they have used to labe the cells.

12.) Fig. 6B and C are redundant - C gives even more information and thus B should be removed from the manuscript.

13.) In Fig. 7+8 authors analyze genes highly expressed in eggs and show that expression is expanded in myb98 mutant ovules - I think they should combine the figures; the phylogram in Fig. 7A is anyhow not very informative and the information can be sufficiently described in the text.

Reviewer #2 (Significance (Required)):

SIGNIFICANCE:

Although this exciting report is descriptive, I am very enthusiastic about the findings: live-cell imaging of FG development is shown for the first time and authors use a large set of markers that can partly be used simultaneously. I expect that their in vitro system will be used by other researchers, and the reporter lines and markers generated will also serve as a solid basis for many future studies by various labs working, for example, on vacuole biogenesis, nuclei positioning, cytoskeleton, signaling etc. Moreover, their description allowed already important conclusions about the timing of cell specification and especially on the fate of the synergid cells. This information will be picked up in future review articles.

In conclusion, I think this is a report with a large impact and of broad interest for the research community, especially for those researchers with a focus on general cell biology and especially

plant reproductive biology. I am also convinced that the manuscript will be often cited for various aspects including methodology, markers, key findings and as a resource of transcriptomic data.

Reviewer #3 (Evidence, reproducibility and clarity (Required)):

This study presents an important advance in the field of sexual plant reproduction. The authors have managed to optimise a system for live-cell imaging of female gametophyte development and cell fate specification using cell specific fluorescent marker lines. Previously, attempts were made to study female gametophyte development by fixing ovules, since live imaging of female gametophyte development was technically challenging due to the process occurring deep within the female pistil. But the authors in the present study used a previously described in vitro ovule culture system, which facilitated studying living dynamics of female gametophytes. They also developed a method to isolate sufficient number of gametophytic cells to perform transcriptome analysis of these individual cells.

In terms of reproducibility, the group is world-leader for this type of microscopy on female gametophytes and therefore the description of protocols and settings used should be as detailed as possible to allow reproducibility. Isolating egg cell and synergid cells from female tissues and then extracting enough RNA to have high quality transcriptome data is also very challenging and many plant labs would find it difficult to do, though it is highly reproducible if done properly.

While the results of this study are very interesting, what it lacks is structure in some parts. There is a redundancy in text, especially in the introduction and in the discussion. Identifying and removing those unnecessary sections that do not contribute to the overall story will improve an otherwise fascinating study.

This study will take us one step closer to a better understanding of female gametophyte development, but some questions remain to be answered or clarified.

Major Comments:

The introduction is too long and too broad. It can be shortened and made more focussed. Some suggestions to improve are given below.

1) Page 2- First column-line 16- How will a reader from a non-plant field understand what is the "chalazal axis"?

2) Page 2- First Column- line 24- Please revise this sentence. Now it sounds like the pollen tube carries only one sperm cell.

3) Page 2- Second column- line 46-47- SC and pollen VN are not protoplasts. Please correct.

4) Page 2- Second column- line 52- Please explain what "greater levels" means.

5) Page 2- Second column- line 54-57- This is an overstatement. We are far from knowing all those genes.

6) Page 3- First column- First paragraph (line 1-19)- This paragraph contributes little to the while story. Consider deletion.

7) Page 3- First column- First paragraph (line 47)- "we then built a technology platform for transcriptome analysis" - standard kits and sequencing technology were used. Consider re-

phrasing.

8) Page 3- Second column - First Paragraph (line 2-12)- Different transgenic lines are described. Are they in Col background?

9) Page 4- First column- line 24-27- How many independent transgenic plants per line were analyzed? And where can the primers be found?

10) Page 4- First column- Section 2.2 Microscopy- Which results were acquired with which system? This should be clarified. Can be a supplemental file.

11) Page 4- Second column- Section 2.3. More detailed are required, for eg: Treated for how long? This paragraph would benefit from more details.

12) Page 4- Second column - Section 2.4- Do these numbers (12-18) apply to all three cell types? RNA-seq experimental method needs to be a bit more detailed, e.g. information on sequencing depth is missing.

13) Page 4- Second column- section 2.5 line 39 - Why were two different analysis methods used?

14) Page 5- First column - First paragraph (line 1-23)- This whole paragraph can be fused with the introduction.

15) Page 5- First column- Line 41-45- How can the authors deduce these specific movements from the videos? How do they know where the "chalazal-micropylar axis" is, and the "surface of the female gametophyte"? In fixed material one could probably define this, but not from the videos shown here.

16) Figure 1B: The authors need to provide a way to identify different stages. It is very difficult to follow the development process based on the images. One solution could be to merge Figure 1A and Figure 1B and show a cartoon with microscopic images.

17) Page 5- First column- line 52- It would help to mention what these markers mark.

18) Page 5- First column - line 53- N=1 is insufficient.

19) Page 6- First column- line 29- Have the authors considered that the promoter sequence might not be complete and regulatory sequences are missing in this construct? Expression of MYB98 might also be regulated by regulatory regions in introns of the genes or in the UTR (both missing in this construct?). What about in situ hybridization? Or are there any transcriptome data for megagametogenesis for this stage that would corroborate these observations? Another important question would be in how many independent transgenic lines this was observed - MYB98pro might drive GFP expression here due to an effect of the integration site into the genome.

20) Page 6- First column- line 36- I do not see this increase. If anything, a decrease in synergid cells. If this claim is to stand, measurements of GFP might be needed.

21) Page 6- First column- line 41- The authors use RPS5Apro::tdTomato-LTI6b construct. Why? Some explanation would help here.

22) Page 6- Second column- line 30- The authors claim "the expression of NLS-mRuby2 was slower than that of the free GFP". It can also be possible that the insertion points into the genome have an influence on expression timing. How many independent transgenic lines were analysed? Page 6- Second column- line 46- There seems to be a control missing here: Measurements in the line MYB98pro::GFP, MYB98pro::NLS-mRuby2 in wild type. Does this one behave as wild type (as

one would expect) or like what is observed in myb98 mutant? In the latter case expression of MYB98pro::GFP would cause the observed phenotype.

23) Page 7- First column- line 23- Why are the synergid cells so large in this image? And the size of the scale bar is missing in the legend.

24) Page 7- Second column-line 3 - The authors should consider introducing the result of DGE here, instead of mentioning it only in the discussion.

25) Page 7- Second column-line 1 and 2- The results of the PCA do not support the claim that "The expression profile of the synergid cells in the mutant was more like that of the egg cells than the synergid cells in the wild type". This needs to be explained better.

26) Page 7- First column-line 51-52- The authors used read count (>10) to detect expressed genes, which is problematic. Since no sequencing depth is given, it is hard for a reader to follow. Normalised values like CPM, TPM or FPKM would be better.

27) Page 9- First Column- line 45- The authors write "A previous microarray analysis of myb98 presented different results," but do not mention what were those previous results and how were they different from what they see in this study. Also, the supplemental file with all tables needs to be arranged properly as it is too difficult to navigate and look for specific DGE results.

28) Page 9- Second column- line 33- The authors mention that "This result indicated
that the synergid cells with the egg cell-specific genes were not functional for fertilization in the myb98". Is it possible that mutant pollen was affected?

29) Page 9- Second column-line 30-33- Can the authors please point to the specific results they are referring here?

30) Page 10- First column- The first paragraph would benefit from extensive re-writing to clarify what the authors want to convey here. The discussion on nuclei positioning and its role in initiation of synergid cell fate needs to be explained properly. Also, the role of AGL80 seems to be misplaced in this part of the discussion. The last line (line 23-25) of this paragraph reading "These findings suggested that the MYB98 may also play a role in preventing the acquisition of egg cell fate in the accessory cells" is not clear at all and needs re-writing to be coherent with the first part of this paragraph.

31) Page 10- First column- line 49. The authors write "that the synergid cells detect the abnormal conditions of the egg cell". Please explain better.
Additionally, as authors mention in several parts of results and discussion, synergids in myb98 mutants behave like egg cell with high expression of egg cell specific genes, such as CDR1L2. What is missing is the information on expression of synergid specific gene markers and how those are affected, e.g. AtLURE1.

Minor Comments:

1) Page 1- Second column-line 6- It remains unclear what the authors want to convey in the second part of this sentence.

2) Page 5- Second column- line 43 - Supplementary Movie 3, "later". Is it not possible to say exactly when?

3) Page 6- First column- line 16- what does "ovules of synergid cells" mean?

4) Page 6- Second column- line 5- Avoid using time points like 1.7 h for example. Please be consistent with hour and time notation. Preferably use 1 h 15 min or 80 min.

5) Page 7- First column- line 40- What were the optimized conditions for these cell types?

6) Page 7- First column- line 54 Figure 6B- Why are the numbers of replicates for samples prepared for this study different?

7) Page 8- Second column-line 21- What does placenta mean and what additional conditions were determined?

8) Page 9- Second column- line 29- "What each gene senses"? It is not clear what the authors want to say here.

9) Figure Legends should be thoroughly checked. For example: Figure 1C, Figure 6A-C.
The contrast of letters within figure needs to be enhanced (For example: Fig. 2C where EC in blue is almost invisible). Similarly, Figure 9, yellow arrows are invisible.

10) Although the authors mention that English editing services were used, the manuscript needs to be improved significantly as far as English is concerned.

For example, the word "the" is used far too often (see for example page 5- First column- line 2) - this needs to be corrected.

Page 4- Second column- line 50- "occurs" and no comma before.

Page 5- First column- line 4- "divides", present tense has to be used here, because it is a general description of the sequence, not something the authors actually observed.

Page 6- First column- line 48- Typo in fig3D: "completion" and not "compleation"

Page 6- Second column- line 43- "it" - should not be "they"?
Page 8- First column- line 28- delete "each"

Page 10- Second column- line 3- not "suggested" but suggest

Page 10- Second column- line 8- "to the gametes" should be "to gamete cell fate"

Page 10- Second column- line 13- Present tense- propose not proposed

Reviewer #3 (Significance (Required)):

This study contributes significantly to a better understanding of plant reproduction. While we know a lot about male gametophyte development, knowledge about female gametophyte development is still limited because of the challenges associated with isolating these specific cells embedded deep within the female tissues. The authors also present future perspectives on their work, like to understand whether synergids acquire the fate of egg cell completely or if they behave partially as egg cell or synergids (and how that would influence pollen tube guidance). In addition, which molecular mechanisms underlie cell to cell communication, is another open question well worth exploring using mutant synergids.
