# Supplementary material for: Dynamics of the cell fate specifications during female gametophyte development in Arabidopsis
Source: PLoS Biol. 2021 Mar 26;19(3):e3001123. doi: 10.1371/journal.pbio.3001123 (PMC7997040; doi:10.1371/journal.pbio.3001123)
Supplement: Attachment — Submitted filename: Susaki_et_al_Response_to_Reviewers.pdf [file pbio.3001123.s039.pdf]

Point-by-Point Response to Reviewer #1

Major comments

We thank you for your kind reviewing and helpful comments. As with your advice, we improved our manuscript with the following points.

Point 1

1) In wild type they note early detection of MYB98pro::GFP throughout the gametophyte prior to cellularization and rapid activation of the EC genes in the newly formed egg cell. However, an important caveat is in the pre-cellularized state (the coenocytic gametophyte), the movement of free GFP, and that of NLS-mRuby2 prior to nuclear import, are both likely and possible respectively. Thus, marker expression will not necessarily mirror localization of MYB98 protein prior to cellularization. Moreover, the longevity of the relatively stable fluorescent proteins could lead to the appearance of expression in newly formed egg and central cells.

Response: As Reviewer#1 comment, that's certainly a possibility. As mentioned in another comments, we observed the ovules of *MYB98pro::GFP–MYB98* as below: "The fluorescent signals of the *MYB98pro::GFP–MYB98* were detected in not only the synergid cells but also the egg cell and the central cell just after cellularization. As the cells mature, the GFP signals were decreased in the egg and central cells while remained in the synergid cells."

Point 2

2) They find that the position of nuclei in the synergids after cellularization is not maintained in myb98 mutants and nuclear positioning becomes more like that in the egg cell. This leads to a muddled message about the role of nuclear position leading to cell fate (and gene expression) vs. gene expression leading to nuclear position: Nuclear position regulates MYB98 initiation but, in myb98, nuclei move around; later MYB98 is still expressed despite this nuclear movement. This apparent contradiction should be more clearly discussed and possible explanations provided.

Response: As shown in Figure 4B, the nucleus moves around the center of the synergid cells in *myb98* mutant. Therefore, we believe that *myb98* synergids can express the specific genes of both egg cell and synergid cell. mSY forms a separate cluster from EC and SY in the PCA analysis (Fig. 6C), suggesting that this possibility. We discussed this possibility as follows: "In the case of *myb98* mutant, the vacuoles were dynamic, causing the nuclei to move around and not to stay in one place (Figure 4B,C). It is considered that this nuclear movement promoted the expression of the egg cell markers in the synergid cells of *myb98*. The gene expression analysis showed the intermediary state between the synergid and egg cell in the *myb98* synergid, suggeting that this nuclear movement may appear as a mixture of the egg and synergid cells identity (Figure **??**B,C,D). Strong correlations between the nuclear position and the cell fate were shown in several mutants (Kong et al., 2015; Groß-Hardt et al., 2007; Pagnussat et al., 2007; Moll et al., 2008; Kirioukhova et al., 2011) However, it remains unclear whether the nuclear position determines gene expression or gene expression determines the nuclear positioning. Manipulation of nuclear behavior with the *in vitro* ovule culture systems will help to reveal the mechanisms of cell fate specifications in the development of the female gametophytes."

Point 3

3) The role of MYB98 in cell fate is investigated by comparing wild type and mutant synergid transcriptomes and cell type marker expression. The authors show that some egg-specific genes are expressed in synergids using transgenes. They conclude from their transcriptomic analysis that mutant synergids are more similar to egg cells.

a. Their RNAseq analyses are not sufficiently well described for full understanding of the results, as detailed in minor comments below. Importantly for low-input RNA libraries, the authors should describe the correlation between replicates statistically, and strategies for overcoming amplification error.

Response: We added the detail in RNA-seq libraries and described the correlation between the replicates in Table S6 and Figure S3A.

b. It appears that their experiments showed both egg and central cells genes are expressed in mutant synergids. From Figure 6A: 225 egg specific genes are found in the myb98 synergid (22% of egg specific genes). However, there are an even greater number of central cell genes mis-expressed in mutant synergids: 838 central cell genes that are turned on (15% of central cell specific genes). Even when only DEGs (SY vs mSY) are used (Figure 6D) and from looking at Table S3 (21 egg genes vs 17 CC genes), there are clearly a comparable number of CC genes expressed in mutant synergids. These central cell expressed genes are not sufficiently addressed in the text. The authors choose to test only egg cell genes by promoter-reporter constructs. The somewhat closer clustering of mutant synergids with egg cells is an indication that overall the cells are more egg cell-like but if that is the most important piece of evidence there should be more details about how the analysis was done. Given the other points above, other interpretations should be considered.

Response: As other reviower's suggest, we deleted the dendrogram for the hierarchical clustering analysis. PCA analysis also indicate the the intermediary state of the *myb98* synergid. We show the gene expression of highly expressed 50 DEGs in egg cells and central cells in the Figure S4. Highly expressed genes of *myb98* synergid cells were more abundant in DEGs of egg cells than those of central cells. Therefore we focused on the egg cell-specific genes.

c. There is little discussion of the actual genes that are changed in expression in the myb98 mutant. Do these provide any evidence of acquisition of cell fate, such as increased expression of egg-cell transcription factors (RKD family) or other regulation?

Response: In our study, RKD1 and RKD2 and RKD2–induced genes were identified as egg cell DEGs, *myb98* DEGs (Köszegi et al., 2011). We confirmed the expression levels of them in textitmyb98 synergid cells. RKD1 and RKD2 were upregulated in a replicate of *myb98* synergid cells. RKD2–induced genes tended to be up–regulated in *myb98* synergid cells. Moreover, EC1.5 was detected as a DEG in *myb98* synergid cell. Other EC1s also tended to be up–regulated in *myb98* synergid cells. We improved the manuscript including above information.

Point 4

4) The authors state that the maintenance, not initiation, of synergid genes was defective in myb98. However, they only give early expression information for MYB98, and note that the synergids were

phenotypically abnormal prior to maturity. No other synergid genes were examined to observe their expression dynamics. No discussion is made of synergid specific genes that were unaffected in the RNAseq of mutants. Some egg genes were observed just after cellularization, indicating an early change from synergid to egg-like. The conclusion that synergids are initiated correctly do not appear to be supported by the data presented.

Response: As Reviewer#1 comment, we only observed the MYB98 expression for the synergid specific genes. We intended that the initiation of synergid cell fate was unaffected, not that the initiation of expression of each synergid gene was unaffected. The subheading "The maintenance, not initiation, of synergid specific genes were defective in myb98" is misleading. So we deleted this subheading and described as below: "The promoter activity of *MYB98* was normal in the *myb98* (Figure **??**B) also suggested that the maintenance, not initiation, of the synergid cell fate were defective in *myb98*".

Point 5

5) MYB98pro::reporter signals were observed throughout the gametophyte prior to and just after cellularization. The expression of one of three examined egg-specific genes (SBT4.13) was observed in antipodals 30% of the time. In contrast, two other genes were observed only in egg and synergid cells. From this, the authors suggest MYB98 negatively regulates egg cell fate in both synergids and antipodals.

a. As mentioned in the first comment, the authors do not account for the movement and persistence of fluorescent reporter proteins which means the true presence of MYB98 protein in antipodals is uncertain. A translational MYB98-GFP fusion should be used if the authors wish to conclude function from reporter expression.

Response: As a response to Point 1, we observed MYB98pro::MYB98–GFP.

b. The number of examined ovules in Tables 1 and 2 (from 6 to 23 ovules) is much lower than typically used to determine trends in gene expression. If these are live imaging experiments then from each experiment, there might be multiple observations of the same ovule, which provides more confidence than a single observation, and might be the reason for the lower number observed. However, to be convincing on the specific points regarding gene expression in the mature FG6 gametophyte in the WT and mutant ovules, a more conventional analysis with greater numbers of observed ovules (n>50) is recommended.

Response: 6 to 23 ovules in Tables 1 and 2 mean different ovules, not the same ovules from live imaging experiments. As Reviewer#1 recommendation, we tried to perform the conventional analysis by observing the ovules from emasculated pistils. But, it was difficult to count the number of synergid cells with egg-cell specific markers by observing the mature ovules. So, we added the data of live imaging experiments (14 to 44 ovules from two to four independent transgenic lines) in Table 1.

Point 6

6) The authors state that there is usually conversion of only one of the synergids to an egg-like state and that this is similar to the known synergid-to-egg conversion when the egg is defective. The authors conclude that this indicates synergid-to-synergid communication. The evidence shown does not make clear that this is the case.

a. For CDR1-L2 and SBT4.13 markers, figures 7C and 8B do not show single synergids, and the information in Table 2 is insufficiently explained to be understood. If these are the same observations as described in Table 1 - what makes the two Tables different is quite unclear (aside from the obviously different numbers in each sub-category). If single synergids are being counted, then shouldn't the total should be double?

Response: We apologize that the label of Table 2 is a mistake. To better understand, we combined Table 1 and Table 2.

b. It is not stated how many times the EC1.1pro expression (Fig8C) was observed.

Response: We observed ten ovules of this line. Five ovules (50 %, n = 10) showed EC1.1pro expression in the synergid cell and remained five ovules showed WT-like.

c. Finally, even if this were clear, a simpler explanation would be that once the synergid acquires some egg-like characteristics, this may include the ability to signal to neighboring synergids to prevent another egg from developing as has been described for egg cells. In other words, what is being observed is another manifestation of the previously described egg cell to synergid communication, rather than synergid to synergid communication.

Response: There is certainly the possibility of communication from the newly egg cell to the synergid cell. In this case, it is necessary to acquire the egg cell characteristics, and there is a time lag before communication occurs. Because some egg-cell specific genes were detected just after cellularization, there should be more cases in which the fluorescent signals of EC-specific genes were detected in the two synergid cells simultaneously. So we think there is also communication between the synergid to synergid cell. We also discussed the possibility of communication from newly egg cell to synergid cell in the revised manuscript as follows: "The results presented here raise two possibilities. One possibility is the synergid cell acquired the egg cell fate, preventing the remaining synergid cell from obtaining the egg cell fate. However, we observed the egg-cell specific genes expressed in both synergid cells in only a few cases (11%, n = 55). In this case, the inhibition signal needs to be quickly transmitted to the remained synergid cell. The other is the cell–cell communication between the two synergid cells to decide which cells convert to the egg cell fate. Exploration and imaging of signal molecules involved in these cell-cell communications would help to reveal these possibilities."

Minor comments

Point 1

1) All figures and Supplemental movies It is important for each time-lapse experiment to clearly state in the legend or text the number of cells observed for each construct.

Response: We provided the sample size in the legend and supplemental table S3.

Point 2

2) Results 3.1 Figure 1A - the model of nuclear movement does not match that described in the text, which is shown in Figure 9. This is confusing to the reader. Figure 1A could be replaced with a simpler version that only shows the stages and not nuclear movements or planes of division. The legend has C mislabeled as B.

Response: Considering the comments of all Reviewers, we removed Fig. 1A.

Point 3

3) It would useful for the authors to compare the findings from Arabidopsis with their prior results on nuclear movement from Torenia (Susaki et al., 2015).

Response: As Reviewer #1 advice, we improved the discussion about the nuclear movement to compare with the previous study in *Torenia*.

Point 4

4) Results 3.3 and beyond There is a bewildering array of promoters and reporters which make the manuscript very difficult to follow, especially as some of the promoters are from genes that are somewhat obscure. It is highly recommended that the authors provide a table to show different promoters, markers, cellular localization and their previously known stages of detection.

Response: We made the marker lines list as a Supplemental Table S1.

Point 5

5) Line 15/16 "in the ovules of the egg cells and the MYB98pro::GFP ovules of the synergid cells" This sentence needs editing, as the meaning is not clear.

Response: We modified the sentence as follows: "We observed the egg cell marker, EC1.2pro::mtKaede (Hamamura et al., 2011), and the synergid cell marker, MYB98pro::GFP (Kasahara et al., 2005) (Figure 3A,B, Supplementary Movie 5, 6)".

Point 6

6) Line 24 ABI4 needs reference

Response: We described the reference for ABI4 (Wang et al., 2010) in the Supplemental table S1 of marker list.

Point 7

7) Figure 3B The black/white pictures of Myb98::GFP are somewhat fuzzy, and definitely not as clear as the colored images with the other marker.

Response: We removed Fig. 3B as we observed *MYB98pro::GFP–MYB98*.

Point 8

8) Figure 3E and Movie S8 There is a missing marker not noted on the figure and movie (purple sporophytic, not AKV), and AKV not labeled in Figure. The use of the non-contrasting sporophytic background marker makes this experiment hard to see clearly.

Response: To make a highly expressed nuclear marker in female gametophyte, 5' UTR of AtADH (Alcohol dehydrogenase) was inserted between AKV promoter and H2B coding sequence. Unfortunately, this AKVpro::H2B-mScarlet-I was expressed not only in female gametophyte but also in sporophytic cells. We described the construct in the material and methods.

Point 9

9) Results 3.4 Please indicate the number of observations made. Figure 4 should be all in color. If equivalent pictures of MYB98pro::GFP/NLS-Ruby in WT are available these should be used, as it is difficult to see the extent of the synergid in A.

Response: As Reviewer #1 recommendation, we changed Fig. 4 as the colored images. We tried to transform the MYB98pro::GFP, MYB98pro::NLS–mRuby2 in Col. Unfortunately, we could not obtain the transformants expressing both MYB98pro::GFP and MYB98pro::NLS–mRuby2 in Col. We added the graph of synergid cell length in wild-type and *myb98* as Supplemental Figure S1.

Point 10

10) Line 50 states that the myb98 synergids were more elongated but it is not possible to see this from the figure, nor are any other measurements given.

Response: We showed the measurement of longitudinal length in the wild-type and *myb98* synergid cells of Supplemental Movie 9 and 10 as Supplemental Figure S1.

Point 11

11) Results 3.5 Figure S1A. A bright field image is needed to show that there are no attached cell debris or other cells.

Response: We don't have the bright field image for previous Fig S1A. Instead, we added the bright field images to Fig 5.

Point 12

12) Figure S1 B legend reads: "F test of frequency showed that there is no significant difference between the two variances ($p > 0.05$)." However, the figure has a single asterisk between the two columns which should indicate that there was a significant difference. RNAseq: With such few cells (12–18) it is important to show that reps are consistent, which was partially done using the PCA and clustering. However, more details are needed, including a correlation analysis. Did the authors use methods to reduce PCR duplicates which would be common from low-input libraries? How did the datasets compare with other cell-specific datasets (Schmid, 2012 and Zhou, 2019). Was there evidence of contamination between cell-types by looking at TPM of genes with known expression patterns?

Response: F-test is the process of checking if the variances of the two groups are equal. First, we confirmed by F-test that the variances of the groups with and without calcium nitrate were equal ($p > 0.05$). Next, Student's t-test showed the absence of calcium nitrate was more effective for synergid cell isolation (*$p < 0.05$). As a response to Point 3 in major comments, we would add the detail in RNA-seq libraries and describe the correlation between the replicates.

Point 13

13) Table S3 and S4 need more explanatory legends for the meaning of the columns, and for S4 how the egg specific genes were selected.

Response: We improved the legend of Table S1–S4.

Point 14

14) Figure 6. The legend has incorrect labeling of A/B/C/D. In B what is the meaning of the scale shown?

Response: We corrected the legend as follows: "RNA-seq of the female gametophyte cells. The biological replicates were sequenced for the two SY, two CC, three EC, and four mSY cells. **(A)** Venn diagram of the expressed genes (> 10 reads) in each cell type. **(B)** Hierarchical clustering of samples for the RNA-seq. Scale bar means height. **C** The PCA analysis of all transcriptome data, the female gametophyte cells, ovules and seedlings. **D** Heatmap of the differentially expressed genes between the synergid cells in the wild type and the *myb98* mutant. OV, ovule; SL, seedling. We improved the method in the manuscript for the hierarchical clustering analysis as follows: Hierarchical clustering analysis according to the value of the Spearman correlation between different samples was carried out using phylogram package (`https://github.com/rambaut/figtree/`).".

Point 15

15) Results 3.6 CDR1-L2 was identified as a myb98 DEG in their analysis, while SBT4.13 and EC1.1 were not, though this is not noted. Why do the authors think this is the case?

Response: In our current thresholds of DEG detection, SBT4.13 and EC1.1 were not detected as *myb98* DEGs. We confirmed the expression levels of SBT4.13 and EC1.1, which had been reported as the egg cell–specific genes. Since the expression of these genes was higher in synergid cells of *myb98* mutant than in those of wild-type, we generated a reporter lines.

Point 16

16) Line 50 - Bleckmann and Dresselhaus 2016 is referenced to indicate that SBT4.13 was known to be mis-expressed in myb98 mutant. However, this reference does not appear to contain any observations on the myb98 mutant. The authors should check this and correct the reference if needed.

Response: We made a mistake. We corrected the sentence as follows: Previously, SBT4.13 were found for the egg cell-specific gene (Bleckmann and Dresselhaus, 2016).

Point 17

17) As mentioned in the major comments, the differences in numbers between Tables 1 and 2 is unclear as they are clearly derived from the same dataset. A clear explanatory legend is needed, and they could be combined if possible.

Response: As a response above, we mistook the label of Table2. We corrected it.

Point-by-Point Response to Reviewer #2

Major comments

We thank you for your kind reviewing and helpful comments. As with your advice, we will improve our manuscript with the following points.

Point 1

1) There are a number of minor issues that should be improved (see below), but most important would be a deeper analysis of their transcriptomic data. Their data is unique, but authors did not spend much efforts to comprehensively report, for example, on genes enriched in the four cell types analyzed. Authors speculate, for example, about synergid-synergid-egg cell communication: thus it would be interesting to know whether genes encoding secreted peptide genes are preferentially expressed in the cell types or whether certain transcription factors, which are often associated with cell fate determination, are specifically expressed. Moreover, the heat map (Fig. 6D) shows DEGs, but we don't learn their identify nor anything about the biological processes that are different between the cells; e.g. do the cells vary in the cell cycle? Synergids are likely in phase G0, while eggs and central cells are supposed to be quickly activated after gamete fusion - but are they similar in this respect? Do synergid-enriched genes especially encode CRPs like in Torenia and which genes are specifically enriched in central cells? I think such bioinformatic analyses would further enhance the significance and impact of their report; these analyses are quickly done and thus, I am a bit surprised that such information is not included in the manuscript.

Response: We added the gene ontology analysis to characterize each cell and make some new figures.

Point 2

2) Another issue is that there is a lot of redundancy in the Discussion; results and constructs are described again, authors refer to images and figures, and partly arguments mentioned already in the Results section are picked up again - I suggest to the authors that they may consider to combine Results and Discussion

Response: As Reviewer #2 suggestion, we combined Results and Discussion to reduce redundancy.

Minor comments

Point 3

3) I think it is a bit strange that almost the same scheme shown in Fig. 1A is again shown in the summary figure Fig. 9; Fig. 9 is very detailed and nicely summarizes major findings of the manuscript and is slightly different to Fig. 1A, which probably aims to indicate the knowledge before this report - however, we know already more than what is shown in Fig. 1A like the formation of a second vacuole at the chalazal end of the FG at stage FG3/4. Thus, my suggestion is to remove Fig. 1A and instead draw the outlines of FG stages in Fig. 1B - I think that's sufficient as this part of the manuscript is accompanied by a number of movies.

Response: As Reviewer #2 comment, we deleted Fig. 1A.

Point 4

4) With the exception of Figs. 5, 6 and 9 titles are missing in figure legends.

Response: We added the titles of figures as follows:

Figure 1: Live-cell imaging of nuclear dynamics during female gametophyte development in *Arabidopsis* using an *in vitro* ovule culture system

Figure 2: Live-cell imaging of cellularization and maturation during FG4–FG5

Figure 3: Live-cell imaging of cell fate specifications during FG4–FG5

Figure 4: Nuclear dynamics in the synergid cells of *myb98*

Figure 7: Expression of EC–specific genes in the *myb98* synergid cells

Point 5

5) In botany the definition of the egg apparatus is "A group of three cells, consisting of the egg and two synergid cells, in the micropylar end of the embryo sac in seed plants." It does not make sense to include the central cell as part of the egg apparatus as done by the authors; the central cell is also fertilized and considered by many researchers as a second female gamete.

Response: We apologize the incorrect description. We improved the introduction and deleted the sentence.

Point 6

6) In the description about RNA-seq in the Introduction authors constitute that rice and Arabidopsis data have been generated using LAM; this is partly not correct and authors should mention that - similar to their study - gametes from rice, maize and Arabidopsis were manually isolated after enzymatic treatment (e.g. rice: Anderson et al., 2017; maize: Chen et al., 2018; Arabidopsis: Zhao et al., 2019).

Response: We apologize for the grammatical mistake to express the cell collected by LAM is only the central cell in Arabidopsis. We improved the introduction and deleted the sentence.

Point 7

7) I don't think authors introduced the LTI6b marker - does it label the whole plasma membrane or only locally? It appears that the membrane at the micropylar pole was not labelled.

Response: The whole plasma membrane was labeled with LTI6b–tdTomato. Depending on the line, only the plasma membrane of female gametophyte cells was labeled, not sporophytic cells, in some RPS5Apro::tdTomato–LTI6b lines. We think the difficulty in seeing the membrane at the micropylar pole is due to the strong signal of the filiform apparatus.

Point 8

8) Authors conclude that the NLS marker was slower detectable compared to the mitochondrial marker; however, they have used different promoters: EC1.1 and EC1.2 - perhaps the genes are activated differently after cellularization - this should be discussed; I am also surprised that many figures involve EC1 promoters/genes which were described in Sprunck et al. 2012, but this paper is not mentioned.

Response:

NLS marker was also slower detected in the MYB98 promoter case, so we believe NLS marker was slow. But as Reviewer #2 says, there is a possibility that the expression of EC1.1 and EC1.2 genes is different. We discussed about the possibility and also cite Sprunck et al. (2012) appropriately as follows: "*EC1.1* and *EC1.2* genes encode the cysteine-rich proteins, are specifically expressed in the egg cell in *Arabidopsis* (Sprunck et al., 2012). It is possible that *EC1.1* (At1g76750) and *EC1.2* (At2g21740) are activated differently. Recently, Eason et al. developed a sensor for transiently expressed proteins (STEP) to detect protein expression rapidly in *E. coli* (Eason et al., 2020). The fluorescence of a dim GFP (gSTEP) increased 11-fold by binding to STEPtag within seconds. It would be useful to analyze the expression timing of gene/protein *in vivo*."

Point 9

9) In the text authors first mention Fig. 5B and then Fig. 5A - this should be switched, either in the text or in the figure.

Response: We improved the sentence as follows: "We treated the ovules in emasculated ovaries of the transgenic marker line for the synergid cells, *MYB98pro::GFP*, with enzyme solutions(Figure Fig 5A). "

Point 10

10) Page numbers are missing for most references.

Response: We added page numbers of references appropriately.

Point 11

11) Fig. 5: In the legend authors should indicate the marker lines they have used to labe the cells.

Response: We added the marker lines to the Figure5 legend.

Point 12

12) Fig. 6B and C are redundant - C gives even more information and thus B should be removed from the manuscript.

Response: As Reviewer #2 suggest, we removed the dendrogram of Figure 6B.

Point 13

13) In Fig. 7+8 authors analyze genes highly expressed in eggs and show that expression is expanded in myb98 mutant ovules - I think they should combine the figures; the phylogram in Fig. 7A is anyhow not very informative and the information can be sufficiently described in the text.

Response: As Reviewer #2 suggestion, we moved Fig 7A and 7B to Supplemental Figure S4, and combined Figs 7 and 8.

Point-by-Point Response to Reviewer #3

Major comments

We thank you for your kind reviewing and helpful comments. As with your advice, we will improve our manuscript with the following points and shortened introduction.

Point 1

1) Page 2- First column-line 16- How will a reader from a non-plant field understand what is the "chalazal axis"?

Response: We modified the sentence to understand the micropyle–chalazal axis for a non-plant field as "the micropyle–chalazal (distal–proximal) axis".

Point 2

2) Page 2- First Column- line 24- Please revise this sentence. Now it sounds like the pollen tube carries only one sperm cell.

Response: We modified the sentences as follows: "In flowering plants, two female gametes are fertilized by two sperm cells carried by the pollen tube. The egg cell is one of the female gametes that forms the embryo in the seeds by fertilization with the sperm cell. The central cell is regarded as one of the gametes because it fertilizes the sperm cell, but the fertilized central cell forms the embryo-nursing tissue endosperm in the seed, and it is not inherited in the next generation."

Point 3

3) Page 2- Second column- line 46-47- SC and pollen VN are not protoplasts. Please correct.

Response: We improved the introduction and deleted the sentence.

Point 4

4) Page 2- Second column- line 52- Please explain what "greater levels" means.

Response: We improved the introduction and deleted the sentence.

Point 5

5) Page 2- Second column- line 54-57- This is an overstatement. We are far from knowing all those genes.

Response: As Reviewer #3 suggests, we deleted the sentence.

Point 6

6) Page 3- First column- First paragraph (line 1-19)- This paragraph contributes little to the while story. Consider deletion.

Response: As Reviewer #3 suggests, we deleted the paragraph.

Point 7

7) Page 3- First column- First paragraph (line 47)- "we then built a technology platform for transcriptome analysis" - standard kits and sequencing technology were used. Consider re-phrasing.

Response: We improved the sentence as follows: We then performed transcriptome analysis using a next-generation sequencer for a small number of isolated female gametophyte cells.

Point 8

8) Page 3- Second column - First Paragraph (line 2-12)- Different transgenic lines are described. Are they in Col background?

Response: All transgenic lines are Col background. To clarify, we modified the sentence as follows: "All *Arabidopsis thaliana* transgenic lines were in the Columbia (Col-0) background, and *myb98* mutant has been described previously (Kasahara et al., 2005)."

Point 9

9) Page 4- First column- line 24-27- How many independent transgenic plants per line were analyzed? And where can the primers be found?

Response: We provided the information the number of transgenic lines, trials, and primers as Supplemental Tables.

Point 10

10) Page 4- First column- Section 2.2 Microscopy- Which results were acquired with which system? This should be clarified. Can be a supplemental file.

Response: We also provided the microscope information for each result as a Supplemental Table S3.

Point 11

11) Page 4- Second column- Section 2.3. More detailed are required, for eg: Treated for how long? This paragraph would benefit from more details.

Response: We improved the sentence as follows: "We used an inverted fluorescence microscope (IX-71; Olympus, Japan) equipped with a three-charge-coupled device (CCD) digital camera (C7780; Hamamatsu Photonics Ltd., Japan). Images were acquired using a 40× objective lens (LUCPlanFl 40×, WD = 2.7–4 mm, NA = 0.60; Olympus). We emasculated the flowers at stage 12c (Christensen et al., 1997). Two days later, the unfertilized ovules of each cell marker line were treated with enzyme solution (1 % cellulase [Worthington, USA], 0.3 % macerozyme R-10 [Yakult, Japan], 0.05 % pectolyase [Kyowa Kasei, Japan], and 0.45 M mannitol [pH 7.0]). To collect the target cells during 20 minutes to 1 hour of enzyme solution treatment, we used a micromanipulator (MN-4, MO-202U; Narishige, Japan) and micropipette (Picopipet HR; Nepa Gene, Japan) with glass capillaries (G-1; Narishige, Japan), which were pulled with a micropipette puller (P-97; Sutter, USA) (Ikeda et al., 2011).".

Point 12

12) Page 4- Second column - Section 2.4- Do these numbers (12-18) apply to all three cell types? RNA-seq experimental method needs to be a bit more detailed, e.g. information on sequencing depth is missing.

Response: We added the detail in RNA-seq libraries and describe the correlation between the replicates in Table S6 and Figure S3A.

Point 13

13) Page 4- Second column- section 2.5 line 39 - Why were two different analysis methods used?

Response: TCC was used to identify the DEGs between two groups. TCC+baySeq could detect the DEGs among three groups. We used additional analysis for three group comparison to more accurately classify DEGs in each group.

Point 14

14) Page 5- First column - First paragraph (line 1-23)- This whole paragraph can be fused with the introduction.

Response: Considering the all Reviewers comments,, we deleted this paragraph in the Introduction.

Point 15

15) Page 5- First column- Line 41-45- How can the authors deduce these specific movements from the videos? How do they know where the "chalazal-micropylar axis" is, and the "surface of the female gametophyte"? In fixed material one could probably define this, but not from the videos shown here.

Response: We can see the shape of the female gametophyte as the background signals in the over-enhanced images as follows.

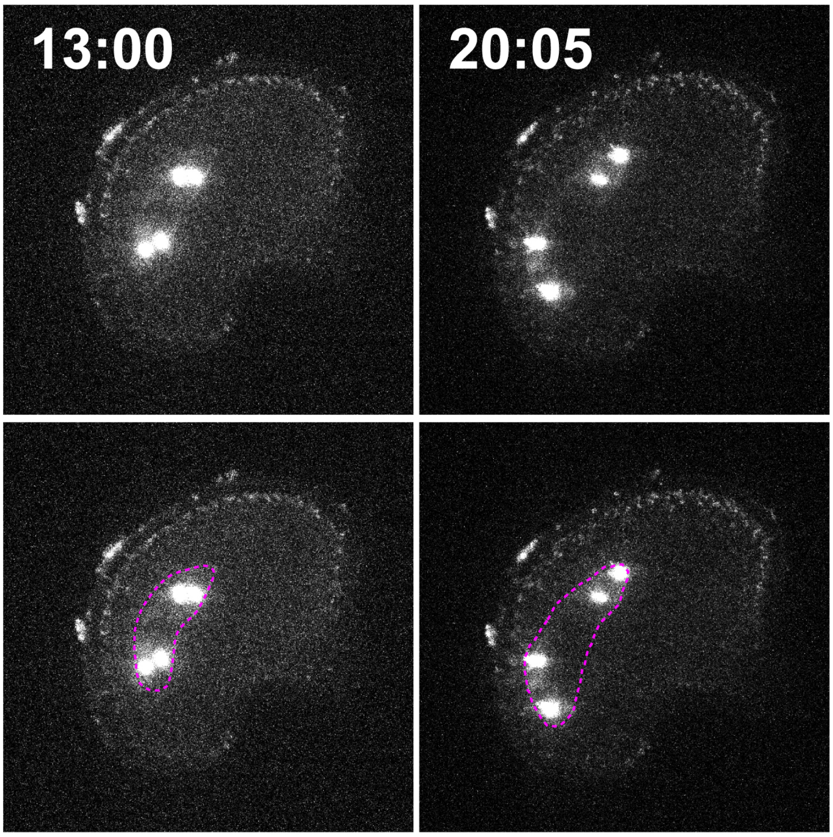

Point 16

16) Figure 1B: The authors need to provide a way to identify different stages. It is very difficult to follow the development process based on the images. One solution could be to merge Figure 1A and Figure 1B and show a cartoon with microscopic images.

Response: Considering the all Reviewers comments, we removed Figure 1A. We identified the FG1, FG2, FG4, FG5 stages by counting the number of nuclei. FG3 was distinguished by the fact that the two nuclei begin to move to both ends. We can identify each mitosis because we can see the chromosomes, not nuclei.

Point 17

17) Page 5- First column- line 52- It would help to mention what these markers mark.

Response: We made the list of each construct as a supplemental table.

Point 18

18) Page 5- First column - line 53- N=1 is insufficient.

Response: We added the data of RPS5Apro::H2B-sGFP (n = 12).

Point 19

19) Page 6- First column- line 29- Have the authors considered that the promoter sequence might not be complete and regulatory sequences are missing in this construct? Expression of MYB98 might also be regulated by regulatory regions in introns of the genes or in the UTR (both missing in this construct?). What about in situ hybridization? Or are there any transcriptome data for megagametogenesis for this stage that would corroborate these observations? Another important question would be in how many independent transgenic lines this was observed - MYB98pro might drive GFP expression here due to an effect of the integration site into the genome.

Response: This MYB98pro::GFP line was established by Kasahara et al. (2005), and there is only one transgenic line. This promoter sequence consists of 1674 bp of sequence upstream of the translational start codon and 72 bp of the first exon of *MYB98*. We have no data on *in situ* hybridization and transcriptome data for megagametogenesis. So, we observed the protein fusion construct (MYB98pro::GFP–MYB98; (Punwani et al., 2007)) as Figure 3B.

Point 20

20) Page 6- First column- line 36- I do not see this increase. If anything, a decrease in synergid cells. If this claim is to stand, measurements of GFP might be needed.

Response: We changed the data of MYB98pro::GFP to that of MYB98pro::MYB98-GFP and described as below:

"As the cells mature, the GFP signals were decreased in the egg and central cells while remained in the synergid cells."

Point 21

21) Page 6- First column- line 41- The authors use RPS5Apro::tdTomato-LTI6b construct. Why? Some explanation would help here.

Response: Depending on the line, only the plasma membrane of female gametophyte cells was labeled, not sporophytic cells, in some RPS5Apro::tdTomato–LTI6b lines. We described it in the revised manuscript. At first, we used this promoter to construct the marker line for female gametophyte imaging. However, we recently found that the GPR1 promoter can label only the female gametophyte cells, so we made a nuclear marker line, but we haven't made a plasma membrane marker line yet.

Point 22

22) Page 6- Second column- line 30- The authors claim "the expression of NLS-mRuby2 was slower than that of the free GFP". It can also be possible that the insertion points into the genome have an influence on expression timing. How many independent transgenic lines were analysed? Page 6- Second column- line 46- There seems to be a control missing here: Measurements in the line MYB98pro::GFP,

MYB98pro::NLS-mRuby2 in wild type. Does this one behave as wild type (as one would expect) or like what is observed in myb98 mutant? In the latter case expression of MYB98pro::GFP would cause the observed phenotype.

Response:

We use a single transformant for this experiment. We have not MYB98pro::GFP, MYB98pro::NLS–mRuby2 in Col., so we tried to transform the MYB98pro::GFP, MYB98pro::NLS–mRuby2 in Col. Unfortunately, we could not obtain the transformants expressing both MYB98pro::GFP and MYB98pro::NLS–mRuby2 in Col. We have observed MYB98pro::GFP and RPS5Apro::H2B–tdTomato, as shown in Figure 3B in the previous manuscript, but the nuclei of the synergid cells stayed at the micropylar end as like MYB98pro::NLS–mRuby2 in Col. So we think MYB98pro::GFP could not cause the observed phenotype of MYB98pro::GFP, MYB98pro::NLS-mRuby2 in *myb98*.

So, we will analyze additional independent lines of MYB98pro::GFP, MYB98pro::NLS–mRuby2 in *myb98* and will need two more months to grow and observe the plants. And now, we try to transform the MYB98pro::GFP, MYB98pro::NLS–mRuby2 in Col., but three months are needed for analysis of T1 plants.

Point 23

23) Page 7- First column- line 23- Why are the synergid cells so large in this image? And the size of the scale bar is missing in the legend.

Response: Scale bar indicate 20 μm. The size of the ovules depends on the size of the plant body. The diameter of the synergid cell protoplasts are about 10 to 20 micrometers.

Point 24

24) Page 7- Second column-line 3 - The authors should consider introducing the result of DGE here, instead of mentioning it only in the discussion.

Response: As Reviewer #3 advice, we improved this paragraph describing about GO analysis for DEGs.

Point 25

25) Page 7- Second column-line 1 and 2- The results of the PCA do not support the claim that ”The expression profile of the synergid cells in the mutant was more like that of the egg cells than the synergid cells in the wild type”. This needs to be explained better.

Response: As Reviewer #3 suggest, we removed the senteence ”The expression profile of the synergid cells in the mutant was more like that of the egg cells than the synergid cells in the wild type”. PCA analysis in Figure 6B and Figure S3B, C indicate the intermediary cell fate state of the *myb98* synergid. Several egg cell-specific genes were highly expressed in the mutant synergid (Figure S4). These results would indicate that the expression pattern of the *myb98* mutant synergid was partially changed to be egg cell-like.

Point 26

26) Page 7- First column-line 51-52- The authors used read count (>10) to detect expressed genes, which is problematic. Since no sequencing depth is given, it is hard for a reader to follow. Normalised values like CPM, TPM or FPKM would be better.

Response: As Reviewer #3 comment in point12, We added the detail in RNA-seq libraries and described the correlation between the replicates in Table S6 and Figure S3A. We improved the data using TPM >1.

Point 27

27) Page 9- First Column- line 45- The authors write "A previous microarray analysis of myb98 presented different results," but do not mention what were those previous results and how were they different from what they see in this study. Also, the supplemental file with all tables needs to be arranged properly as it is too difficult to navigate and look for specific DGE results.

Response:

We described the differences as follows: "Although a previous microarray analysis could not detect the change to the egg cell-like in the synergid cells of *myb98*, this is thought to be because their sample contained the entire ovule, not the synergid cells alone (Jones-Rhoades et al., 2007).". As Reviewer #3 advice, we added some excel sheets for each cell's DEGs.

Point 28

28) Page 9- Second column- line 33- The authors mention that "This result indicated that the synergid cells with the egg cell-specific genes were not functional for fertilization in the myb98". Is it possible that mutant pollen was affected?

Response: If the pollen tube of *myb98* is affected, fertilization with the egg cell as well as the synergid cells with the egg cell-specific genes should be affected. However, the embryo was normally observed in *myb98* mutant, so the effect of *myb98* pollen tube is unlikely.

Point 29

29) Page 9- Second column-line 30-33- Can the authors please point to the specific results they are referring here?

Response: We added the embryo of *myb98* homozygous mutant as a Supplemental Figure S6.

Point 30

30) Page 10- First column- The first paragraph would benefit from extensive re-writing to clarify what the authors want to convey here. The discussion on nuclei positioning and its role in initiation of synergid cell fate needs to be explained properly. Also, the role of AGL80 seems to be misplaced in this part of the discussion. The last line (line 23-25) of this paragraph reading "These findings suggested that the MYB98 may also play a role in preventing the acquisition of egg cell fate in the accessory cells" is not clear at all and needs re-writing to be coherent with the first part of this paragraph.

Response: As Reviewer #3 suggestion, we deleted the discussion about AGL80 and discussed as

follows:

"Previously, it has been reported that *MYB98pro::GFP* is expressed in all cells of the female gametophyte, except for the antipodal cells at FG5 (Ingouff et al., 2006). The fluorescent signals of the GFP–MYB98 and NLS–mRuby2 were also detected in the synergid, egg, and central cells just after cellularization (Figure 3B, 4A). Except for the synergid cells, the fluorescent signals of the GFP–MYB98 and NLS–mRuby2 were decreased as the cells matured. These results suggested that the synergid cell fate stabilized the gene expression of the *MYB98*. The ectopic expressions of the *MYB98pro::GFP* and *MYB98pro::NLS–mRuby2* were not detected after the restrictions of the expression to the synergid cells in the *myb98* mutant (Figure 4B). This suggested that the egg and central cells regularly maintain their cell fates. The promoter activity of *MYB98* was normal in the *myb98* (Figure 4B) also suggested that the maintenance, not initiation, of the synergid cell fate were defective in *myb98*. Considering these results, the positional information of nuclei is essential for the initiation of the synergid cell fate."

Point 31

31) Page 10- First column- line 49. The authors write "that the synergid cells detect the abnormal conditions of the egg cell". Please explain better. Additionally, as authors mention in several parts of results and discussion, synergids in myb98 mutants behave like egg cell with high expression of egg cell specific genes, such as CDR1L2. What is missing is the information on expression of synergid specific gene markers and how those are affected, e.g. AtLURE1.

Response: Considering Reviewer #3 comment, we modified the sentence as follows: "Lateral inhibition from the egg cell restricts the egg cell fate in accessory cells (Völz et al., 2013)). Based on these findings, we speculate that egg-cell failure induce a decrease of *MYB98* expression. The combination of these cell–cell communications and the flexible fate maintenance might allow for only one of the two synergid cells to become an egg cell."

We also added the data and the references about the synergid-specific genes in *myb98* as follows: "The expression of AtLURE1 was also decreased in the *myb98* synergids (Takeuchi and Higashiyama, 2012)"

Minor comments

Point 1

1) Page 1- Second column-line 6- It remains unclear what the authors want to convey in the second part of this sentence.

Response: To better understanding, we modified the sentence as follows: "The development of the female gametophyte in angiosperms is an attractive model for studying cell fate specifications."

Point 2

2) Page 5- Second column- line 43 - Supplementary Movie 3, "later". Is it not possible to say exactly when?

Response: To better understanding, we changed to "second half of Supplementary Movie 3".

Point 3

3) Page 6- First column- line 16- what does "ovules of synergid cells" mean?

Response: We modified the sentence as follows: ”We observed the egg cell marker, EC1.2pro::mtKaede (Hamamura et al., 2011), and the synergid cell marker, MYB98pro::GFP (Kasahara et al., 2005) (Figure 3A,B, Supplementary Movie 5, 6)”.

Point 4

4) Page 6- Second column- line 5- Avoid using time points like 1.7 h for example. Please be consistent with hour and time notation. Preferably use 1 h 15 min or 80 min.

Response: According Reviewer #3 suggestion, we modified time notation as hr:min.

Point 5

5) Page 7- First column- line 40- What were the optimized conditions for these cell types?

Response: Egg cells were detached from ovules same as synergid cells. The frequency of released central cells were lower than that of egg cells. After 30 minutes of treatment with the enzyme solution, ovules without released central cells were gently pressed by glass capillary to help release from the ovules. We added these information to material and methods section.

Point 6

6) Page 7- First column- line 54 Figure 6B- Why are the numbers of replicates for samples prepared for this study different?

Response: Collection of synergid cells and central cells was much more difficult than that of egg cells and synergid cells of *myb98* mutant.

Point 7

7) Page 8- Second column-line 21- What does placenta mean and what additional conditions were determined?

Response: To combine Results and Discussion, we deleted this paragraph.

Point 8

8) Page 9- Second column- line 29- ”What each gene senses”? It is not clear what the authors want to say here.

Response: To better understanding, we modified the sentence as follows: ”These results suggested that the regulations of each egg-cell specific gene expression are different.”

Point 9

9) Figure Legends should be thoroughly checked. For example: Figure 1C, Figure 6A-C. The contrast of letters within figure needs to be enhanced (For example: Fig. 2C where EC in blue is almost invisible). Similarly, Figure 9, yellow arrows are invisible.

Response: We double-checked the figure legend and modify the color to better visibility.

Point 10

10) Although the authors mention that English editing services were used, the manuscript needs to be improved significantly as far as English is concerned.

Response: English editing service checked again with Reviewer #3 comments in the revised manuscript.

References

Christensen, C. A., King, E. J., Jordan, J. R., and Drews, G. N. (1997). Megagametogenesis in Arabidopsis wild type and the Gf mutant. *Sex Plant Reprod*, 10(1):49–64.

Eason, M. G., Pandelieva, A. T., Mayer, M. M., Khan, S. T., Garcia, H. G., and Chica, R. A. (2020). Genetically Encoded Fluorescent Biosensor for Rapid Detection of Protein Expression. *ACS Synth Biol*, page acssynbio.0c00407.

Groß-Hardt, R., Kägi, C., Baumann, N., Moore, J. M., Baskar, R., Gagliano, W. B., Jürgens, G., and Grossniklaus, U. (2007). LACHESIS Restricts Gametic Cell Fate in the Female Gametophyte of Arabidopsis. *PLoS Biology*, 5(3):e47.

Hamamura, Y., Saito, C., Awai, C., Kurihara, D., Miyawaki, A., Nakagawa, T., Kanaoka, M. M., Sasaki, N., Nakano, A., Berger, F., and Higashiyama, T. (2011). Live-cell imaging reveals the dynamics of two sperm cells during double fertilization in Arabidopsis thaliana. *Curr Biol*, 21(6):497–502.

Ikeda, Y., Kinoshita, Y., Susaki, D., Ikeda, Y., Iwano, M., Takayama, S., Higashiyama, T., Kakutani, T., and Kinoshita, T. (2011). HMG Domain containing SSRP1 is required for DNA demethylation and genomic imprinting in arabidopsis. *Dev Cell*, 21(3):589–596.

Ingouff, M., Jullien, P. E., and Berger, F. (2006). The Female Gametophyte and the Endosperm Control Cell Proliferation and Differentiation of the Seed Coat in Arabidopsis. *Plant Cell*, 18(12):3491–3501.

Jones-Rhoades, M. W., Borevitz, J. O., and Preuss, D. (2007). Genome-Wide Expression Profiling of the Arabidopsis Female Gametophyte Identifies Families of Small, Secreted Proteins. *PLoS Genet*, 3(10):e171.

Kasahara, R. D., Portereiko, M. F., Sandaklie-Nikolova, L., Rabiger, D. S., and Drews, G. N. (2005). MYB98 Is Required for Pollen Tube Guidance and Synergid Cell Differentiation in Arabidopsis. *Plant Cell*, 17(11):2981–2992.

Kirioukhova, O., Johnston, A. J., Kleen, D., Kägi, C., Baskar, R., Moore, J. M., Bäumlein, H., Groß-Hardt, R., and Grossniklaus, U. (2011). Female gametophytic cell specification and seed development require the function of the putative Arabidopsis INCENP ortholog WYRD. *Development*, 138(16):3409–3420.

Kong, J., Lau, S., and Jürgens, G. (2015). Twin Plants from Supernumerary Egg Cells in Arabidopsis. *Curr Biol*, 25(2):225–230.

Köszegi, D., Johnston, A. J., Rutten, T., Czihal, A., Altschmied, L., Kumlehn, J., Wüst, S. E. J., Kirioukhova, O., Gheyselinck, J., Grossniklaus, U., and Bäumlein, H. (2011). Members of the RKD transcription factor family induce an egg cell-like gene expression program. *Plant Journal*, 67(2):280–291.

Moll, C., von Lyncker, L., Zimmermann, S., Kägi, C., Baumann, N., Twell, D., Grossniklaus, U., and Groß-Hardt, R. (2008). CLO/GFA1 and ATO are novel regulators of gametic cell fate in plants. *Plant J*, 56(6):913–921.

Pagnussat, G. C., Yu, H.-J., and Sundaresan, V. (2007). Cell-Fate Switch of Synergid to Egg Cell in Arabidopsis eostre Mutant Embryo Sacs Arises from Misexpression of the BEL1-Like Homeodomain Gene BLH1. *Plant Cell*, 19(11):3578–3592.

Punwani, J. A., Rabiger, D. S., and Drews, G. N. (2007). MYB98 Positively Regulates a Battery of Synergid-Expressed Genes Encoding Filiform Apparatus–Localized Proteins. *Plant Cell*, 19(8):2557–2568.

Sprunck, S., Rademacher, S., Vogler, F., Gheyselinck, J., Grossniklaus, U., and Dresselhaus, T. (2012). Egg Cell-Secreted EC1 Triggers Sperm Cell Activation During Double Fertilization. *Science*, 338(6110):1093–1097.

Susaki, D., Takeuchi, H., Tsutsui, H., Kurihara, D., and Higashiyama, T. (2015). Live imaging and laser disruption reveal the dynamics and cell-cell communication during torenia fournieri female gametophyte development. *Plant Cell Physiol*, 56(5):1031–1041.

Takeuchi, H. and Higashiyama, T. (2012). A Species-Specific Cluster of Defensin-Like Genes Encodes Diffusible Pollen Tube Attractants in Arabidopsis. *PLoS Biol*, 10(12):e1001449.

Völz, R., Heydlauff, J., Ripper, D., von Lyncker, L., and Groß-Hardt, R. (2013). Ethylene Signaling Is Required for Synergid Degeneration and the Establishment of a Pollen Tube Block. *Dev Cell*, 25(3):310–316.

Wang, D., Zhang, C., Hearn, D. J., Kang, I.-H., Punwani, J. A., Skaggs, M. I., Drews, G. N., Schumaker, K. S., and Yadegari, R. (2010). Identification of transcription-factor genes expressed in the Arabidopsis female gametophyte. *BMC Plant Biol*, 10(1):110.
