# Supplementary material for: Dynamics of the cell fate specifications during female gametophyte development in Arabidopsis
Source: PLoS Biol. 2021 Mar 26;19(3):e3001123. doi: 10.1371/journal.pbio.3001123 (PMC7997040; doi:10.1371/journal.pbio.3001123)
Supplement: Attachment — Submitted filename: Susaki_et_al_response.pdf [file pbio.3001123.s040.pdf]

Point-by-Point Response to Reviewer #3

We thank you for your kind reviewing and helpful comments. As with your advice, we improved our manuscript with the following points.

Point 1

1) I appreciate the authors' efforts to obtain lines expressing MYB98pro::GFP, MYB98pro::NLS-mRuby2 in myb98 mutant and lines expressing MYB98pro::GFP, MYB98pro::NLS-mRuby2 in Col. wild type, but I do not think the current lack of these lines should prevent publication and cause delays.

Response: We thank you for your kind advice.

Point 2

2) Figure numbers have been replaced with question marks in this current version of the manuscript.

Response: We corrected the figure numbers in the main text of this revised manuscript.

Point 3

3) Page 6, line 50: The GO analysis can be improved. For one, it is not clear how the list of genes underlying the GO analysis was obtained. Which samples exactly were compared to get to the "DEG in EC" list, for example? Secondly, the results of the GO analysis are not really discussed. This might be due to the limited amount of meaningful GO terms identified. The author's might want to give this tool a try instead: https://biit.cs.ut.ee/gprofiler/gost.

Response: As Reviewer #3 advised, we improved the GO enrichment analysis and replaced Figure 6C to Tables 1, 2 and Table S11–13, which were made with the tool recommended by Reviewer #3. In the manuscript, we improved the discussion about the GO enrichment analysis as below:

"Gene Ontology (GO) enrichment analysis of the DEGs that were specifically expressed in the wild type female gametophyte cells indicated that the GO terms of each cell type were related to protein secretion and cell wall synthesis in the synergid cells; fertilization, DNA methylation and small regulatory RNA in the egg cells; and photosynthesis and interaction with other cells (cell killing and defense response) in the central cells (Tables 1, 2, S12–14). These results are consistent with the known functions of each cell type. The synergid cells play a role in pollen tube guidance through the secretion of many peptides, including *AtLUREs*, the filiform apparatus, and thickened cell wall structure (Takeuchi and Higashiyama, 2012). GO terms supported that RNA–based gene silencing and DNA methylation mechanisms have important roles in the egg cells. The central cells play a role in pollen tube guidance through regulating the expression of cysteine–rich peptides (CRPs) from the synergid and central cells (Chen et al., 2007; Li et al., 2015; Takeuchi and Higashiyama, 2012). As in the present study, photosynthesis has recently been reported as a central cells–specific GO term (Song et al., 2020). Including other GO terms, the central cells have high metabolite levels (Table S14)."
